# Computational modeling of tissue damage preceding aortic dissection: a coupled biphasic and reactive viscoelastic framework

**DOI:** 10.1007/s10237-026-02114-1

**Published:** 2026-07-22

**Authors:** Laura Pellerito, Stéphane Avril, Elisabetta Morici, Giuseppe Sancataldo, Massimiliano Zingales

**Affiliations:** 1 Department of Me.Pre.C.C., University of Palermo, Via Liborio Giuffrè n°5, 90127 Palermo, Italy; 2 Mines Saint-Étienne, University Jean Monnet, INSERM, U 1059 Sainbiose, 42023 Saint-Étienne, France; 3 Advanced Technologies Network (ATeN) Center, University of Palermo, Viale delle Scienze, Ed. 18, 90128 Palermo, Italy; 4 Department of Physics and Chemistry, University of Palermo, Viale delle Scienze, Ed. 18, 90128 Palermo, Italy; 5 Department of Engineering, University of Palermo, Viale delle Scienze, Ed. 8, 90128 Palermo, Italy

**Keywords:** Aortic dissection, Biphasic modeling, Reactive viscoelasticity, Radial tensile testing, Damage mechanics

## Abstract

Aortic dissection is a life-threatening pathology characterized by the progressive delamination of adjacent lamellar units within the aortic media. Because this internal damage propagates predominantly along the radial direction of the arterial wall, radial tensile testing has emerged as a particularly relevant experimental configuration to reproduce the mechanical conditions associated with dissection. Several experimental studies have reported the mechanical response of arterial tissues under radial tension, highlighting pronounced viscoelasticity, fluid-driven effects, and progressive damage. However, despite these advances, a coherent constitutive framework capable of reproducing the full mechanical response of arterial tissue subjected to radial tensile loading is still lacking. In this study, we propose a computational model specifically designed to describe arterial tissue behavior under radial tensile testing. The model combines a biphasic formulation, accounting for fluid–solid interactions, with a reactive viscoelastic damage framework to capture time-dependent response and progressive mechanical degradation. Implemented within the FEBio environment, the model is calibrated using experimental radial tensile tests on aortic tissue. The proposed formulation accurately reproduces key experimental features, including stress relaxation, nonlinear stiffening, and damage progression. These results demonstrate that the model provides a physically consistent description of arterial tissue behavior under radial tension and represents a relevant tool for investigating the mechanical mechanisms preceding aortic dissection.

## Introduction

Aortic dissection (AD) is one of the most severe pathologies affecting the aorta, with a high mortality rate if left untreated. It is characterized by mechanical separation and delamination of adjacent lamellar units within the arterial media, leading to the formation of a false lumen parallel to the true lumen. Despite its clinical relevance, the mechanical mechanisms that govern the initiation and propagation of this internal damage remain incompletely understood. From a biomechanical standpoint, aortic dissection is intrinsically a radial failure phenomenon (Mikich 2003). Following an initial tear or defect in the intimal layer–whether due to intrinsic degeneration, intramural hematoma, or iatrogenic injury–localized pressurization induces stresses that act predominantly perpendicular to the arterial wall layers (Khanafer and Berguer 2009; Pasta et al. 2012). This loading configuration promotes damage propagation along the radial direction, ultimately leading to lamellar separation (Baliga et al. 2007). Consequently, mechanical characterization techniques capable of probing the arterial wall response along its thickness are essential for understanding dissection mechanisms (Sommer et al. 2010).

In this context, radial tensile testing has emerged as a particularly relevant experimental approach. Unlike conventional axial or circumferential tensile tests, which primarily probe the load-bearing capacity of collagen fibers aligned with physiological stresses, radial tensile tests directly interrogate interlamellar cohesion and transverse mechanical integrity (Samila and Carter 1981). Experimental studies have demonstrated that arterial tissue exhibits markedly lower stiffness and strength in the radial direction, providing a mechanical explanation for the ease with which dissections propagate once initiated (MacLean et al. 1999; Thubrikar et al. 2001). Moreover, radial tensile loading induces significant volume changes and interstitial fluid redistribution, highlighting the importance of the biphasic nature of arterial tissue under this specific loading condition (Simon 1992). Several experimental investigations have reported the mechanical response of arterial tissue subjected to radial tension, documenting nonlinear behavior, pronounced stress relaxation, and progressive damage (Sommer et al. 2008). Advanced experimental methodologies have further enabled the measurement of local strain fields and microstructural reorganization preceding failure (Giuseppe et al. 2020). These studies collectively establish radial tensile testing as a meaningful surrogate for studying dissection-related damage mechanisms. However, despite this growing body of experimental evidence, a critical gap remains at the modeling level. Existing computational models of aortic dissection primarily focus on macroscopic failure scenarios or idealized geometries and often rely on constitutive laws calibrated from axial or circumferential data (Holzapfel et al. 2000). While some models incorporate progressive damage or fiber degradation (Gültekin et al. 2019; Rolf-Pissarczyk et al. 2021), many neglect the biphasic nature of arterial tissue and its associated fluid–solid interactions (Bukac et al. 2015). Recent literature emphasizes the importance of integrating inelastic phenomena, such as damage and anisotropic viscoelasticity, to accurately predict the physiological and pathological response of soft biological tissues (Holzapfel and Ogden 2025). However, most existing models fail to provide a constitutive framework specifically developed and validated to reproduce the complex mechanical response observed under radial tensile loading.

The objective of this study is to address this gap by proposing a constitutive model tailored to arterial tissue subjected to radial tensile loading. By combining a biphasic formulation with a reactive viscoelastic damage model, the proposed approach aims to capture the coupled effects of fluid transport, viscoelasticity, and progressive microstructural degradation. Calibrated against experimental radial tensile tests, this model provides a physically consistent description of arterial wall behavior under conditions directly relevant to aortic dissection, thereby offering a new computational tool for investigating the mechanical processes preceding this catastrophic pathology. All computational simulations were performed using the open-source software FEBio, which is specifically tailored for the nonlinear mechanics of hydrated biological tissues (Maas et al. 2012; Shim et al. 2021).

## Materials and methods

### Radial tensile testing as a surrogate for dissection-related loading

Radial tensile testing is employed as a fundamental experimental surrogate as it directly replicates the interlamellar separation and transverse failure characteristic of aortic dissection. This specific loading configuration triggers significant interstitial fluid redistribution and progressive microstructural degradation within the medial lamellae (Sommer et al. 2008). Consequently, the implementation of this experimental protocol effectively captures the underlying mechanisms of internal damage propagation that drive the progression toward total aortic wall dissection.

### Integrated research strategy and modeling workflow

**Fig. 1:**
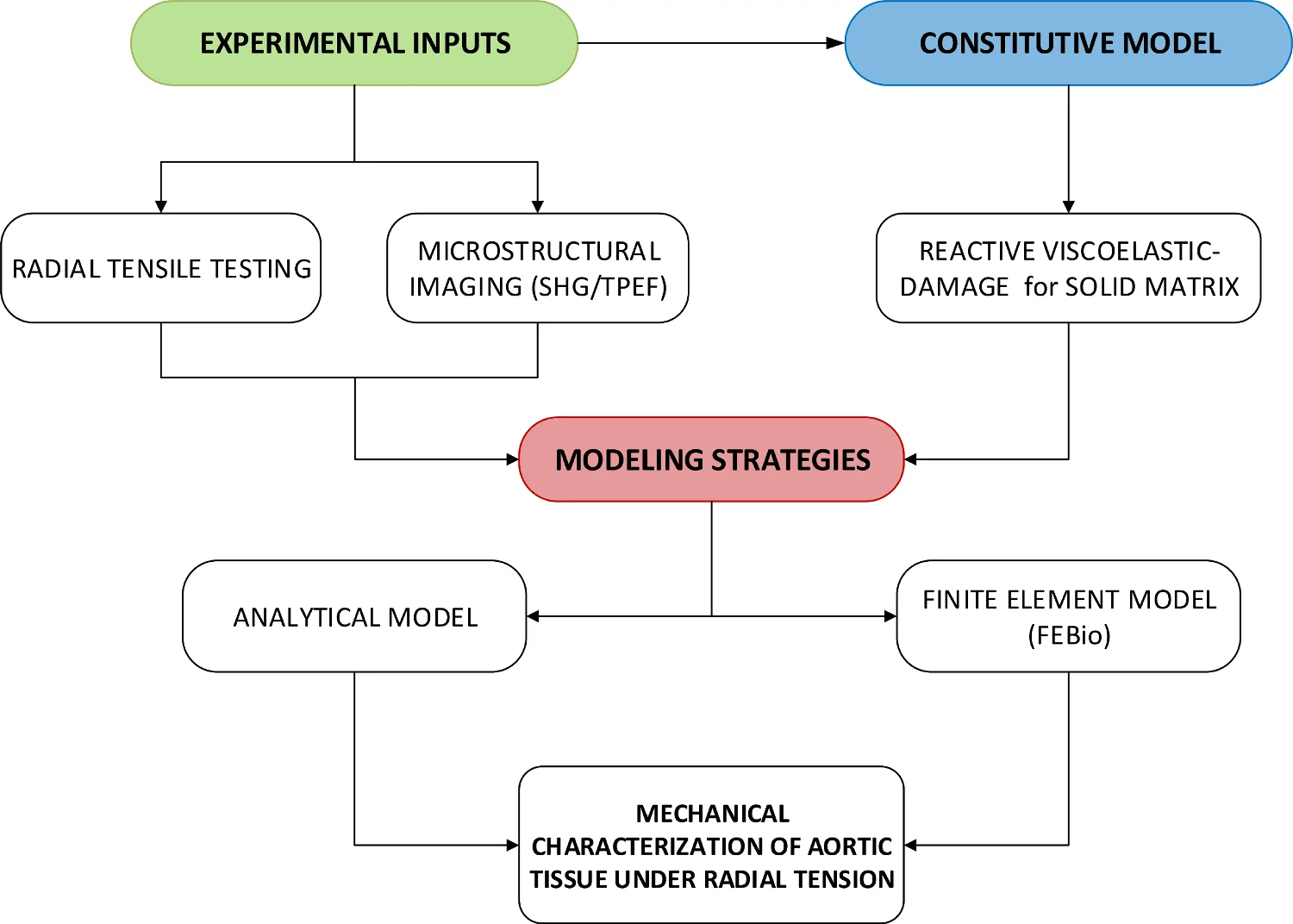
Methodological workflow of the integrated approach. Experimental inputs (radial tensile tests and microstructural imaging) inform the constitutive model of the solid matrix. The framework is deployed through two modeling strategies: an analytical model for parameter identification and a Finite element model (FEBio) for poromechanical validation, leading to the final mechanical characterization of the aortic tissue

The research methodology follows an integrated experimental-numerical workflow designed to bridge microstructural observations with the macroscopic mechanical response, as summarized in Fig. 1. Microstructural and mechanical experimental inputs directly inform the theoretical constitutive model, which is based on a reactive viscoelastic-damage framework for the solid matrix. This theory is implemented through two complementary modeling strategies with distinct objectives. First, an analytical model is calibrated using experimental stress-time data to identify the material parameters governing the intrinsic viscoelastic response of the solid matrix. Subsequently, a finite element model (FEBio software) is developed to validate the total mechanical response, explicitly accounting for the poromechanical coupling between the solid phase and the interstitial fluid flow. This dual-stage strategy is designed to ensure that the identified constitutive parameters accurately reflect the physical behavior of the tissue, rather than purely relying on numerical fitting. By verifying these parameters within a poroelastic framework, the approach aims to characterize the synergistic interaction between the solid matrix and interstitial fluid during the complex stress-relaxation phases observed experimentally.

### Tissue preparation and sample geometry

**Fig. 2:**
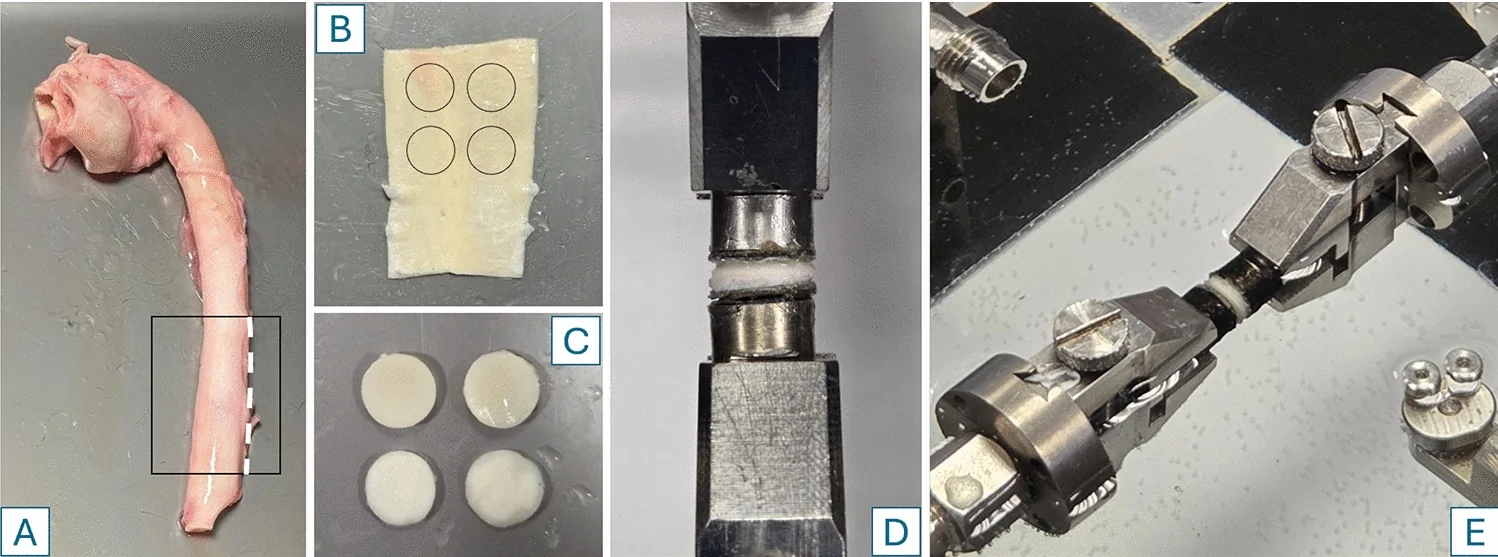
Step-by-step preparation of circular aortic tissue samples: **A** segment of the porcine descending thoracic aorta used for specimen harvesting; **B, C** extraction of four cylindrical specimens (10 mm diameter) from the highlighted region; **D** detailed view of the specimen bonded to the cylindrical metal platens; **E** final experimental setup with the sample mounted on the Bose Electroforce system and submerged in a saline bath at 37 $^{\circ }$C

Six-month-old porcine aortic tissues were collected from a local slaughterhouse. Specifically, segments were isolated from the lower descending thoracic aorta, proximal to the abdominal junction. As shown in Fig. 2A, B, C, multiple 10 mm diameter circular specimens–specifically, four technical replicates per animal—were extracted from each aortic segment at the same anatomical level to ensure regional consistency. Across the cohort of five distinct animals, a total of twenty specimens were successfully harvested for the experimental test. The initial geometry of each specimen was characterized with high precision to ensure accurate stress and strain calculations. Specifically, the radial thickness ($H_0$) was measured using a Sensofar S neox 3D optical profilometer. This non-contact technique allowed for the acquisition of the specimen’s topography without inducing any mechanical pre-compression of the soft tissue. For each sample, the thickness was determined by averaging measurements taken at three distinct locations along the longitudinal axis, reported in Table 1. The resulting mean thickness for the five analyzed specimens was 2.02±0.22 mm (mean ± inter-sample SD), reflecting the inherent biological variability of the aortic media. Based on the uniform 10 mm diameter of the circular cutting punch, the initial reference volume ($V_0$) of each specimen was computed (reported in Table 1) to provide the FEBio numerical model with the correct baseline geometric domain required to solve the coupled fluid-solid poromechanical equations. The variability in wall thickness observed across the specimens reflects a well-known challenge in aortic biomechanics, where measurement uncertainties can significantly impact stress estimation (Smoljkić et al. 2016). All samples were maintained in Phosphate-Buffered Saline (PBS) solution at room temperature until testing to preserve hydration and structural integrity.

**Table 1:** Geometrical characterization of the aortic specimens

Sample ID	Mean $H_0$ (mm)	Volume $V_0$ (mm$^3$)
1	2.18 ± 0.080	171.26
2	1.94 ± 0.078	152.68
3	1.87 ± 0.072	147.18
4	2.31 ± 0.082	181.46
5	1.78 ± 0.085	140.25
**Global Mean**	**2.02 ± 0.22** $^{1}$	**158.56**

$^1$Inter-sample standard deviation representing biological variability

Initial radial thickness ($H_0$) measured via non-contact 3D optical profilometry, and initial reference volume ($V_0$) calculated from the uniform 10 mm nominal punch diameter. The reported parameters provide the high-precision reference geometry required for the numerical poromechanical model

### Experimental tests

Radial tensile tests were conducted in the Laboratory of Mechanics of Materials and BioMaterials of the ATeN Center at the University of Palermo, using a Bose Electroforce test system equipped with dual actuators. Following the identification of the porcine descending thoracic aorta segments (Fig. 2A), four 10 mm diameter cylindrical specimens were extracted for testing (Fig. 2B, C). To ensure a uniform distribution of the tensile load throughout the cross-section of the sample, the specimens were fixed to custom-made metal cylinders of matching diameter (10 mm) using a high-strength cyanoacrylate adhesive (Fig. 2D). Before starting the test, a constant compressive load of 1 N was applied for 5 min to ensure optimal adhesive bonding at the tissue-tool interface. To simulate physiological conditions and prevent tissue dehydration, all experiments were conducted in a saline bath kept at a constant temperature of 37 $^{\circ }$C (Fig. 2E).

First, a sinusoidal preconditioning protocol was applied—10 cycles, 0.1 Hz frequency, 1 mm amplitude—to achieve repeatable mechanical state and minimize tissue hysteresis. Subsequently, a displacement-controlled step-relaxation test was executed, consisting of an instantaneous displacement of 0.082 mm followed by a 30-second relaxation period at constant strain. The choice of a displacement-controlled protocol is fundamental to accurately define the strain state and provide a prescribed input for the subsequent computational calibration. This loading sequence was designed to isolate the time-dependent mechanical features of the aortic wall (Valente et al. 2024). Specifically, the step-relaxation configuration is critical, as it allows for the decoupling of the instantaneous elastic response from the time-dependent dissipative mechanisms. This approach explicitly triggers both the viscoelasticity of the solid matrix and the biphasic effect given the flow of the interstitial fluid, providing the experimental basis necessary to justify and validate the model (Leng et al. 2018). Furthermore, the small displacement magnitude—0.082 mm, corresponding to a strain of $\approx 4\%$—represents an infinitesimal loading regime designed to capture the incipient stages of interlamellar damage. The subsequent 30-second hold allows the internal fluid pressure to equilibrate, enabling the tissue to reach a quasi-static state. This is essential for isolating damage-induced degradation from transient viscous effects, ensuring a consistent transition from experimental observation to computational modeling.

It is worth noting that despite the free lateral boundary of the specimens, short-term lateral deformation is physically restricted during each loading step. Under the rapid displacement ramps initiating each relaxation window, the interstitial fluid is transiently trapped within the interlamellar spaces due to the tissue’s low hydraulic permeability. This fluid entrapment generates an instantaneous hydrostatic pore pressure gradient that resists lateral contraction, causing the tissue to act as a poro-mechanically constrained medium at the onset of relaxation. This transient effect is further reinforced by the dense, stiff in-plane collagen network, which mechanically minimizes lateral necking during out-of-plane radial tension, effectively preserving a localized uniaxial state within each individual relaxation window.

To ensure reliable data fitting and isolate true inter-individual variability, only one representative specimen per animal was selected from the twenty tested samples. This selection was based on a practical quality control of the raw experimental curves: technical replicates were excluded if they exhibited premature tissue rupture, macro-slipping at the glued interface, or anomalous force-displacement profiles due to experimental artifacts. The most consistent, stable, and regular multi-step relaxation curve from each individual was chosen, yielding a final independent calibration dataset of five specimens.

### Microstructural characterization to inform constitutive modeling

To provide a physical basis for the constitutive framework and characterize the damage-induced reorganization of the medial lamellae, microstructural imaging was performed at the Advanced Microscopy and Bioimaging Laboratory of the University of Palermo. Multiphoton microscopy (Leica TCS SP5 confocal laser scanning microscope, equipped with an oil immersion objective (63X, NA 1.40)) was employed to visualize the primary load-bearing components of the aortic wall without the use of exogenous markers. Collagen-derived SHG signal was detected in the range 390–460 nm, while elastin TPEF signal was recorded simultaneously between 485 and 650 nm. Recent advancements have consolidated this label-free approach as a powerful tool for volumetric quantification of the elastin-to-collagen ratio and for assessment of microstructural remodeling in diseased aortas (Pukaluk et al. 2022; Cavinato et al. 2020). This technique is now considered the gold standard for non-destructively characterizing the three-dimensional lamellar organization and fiber recruitment under mechanical loading (López-Guimet et al. 2017), providing the necessary structural priors for advanced constitutive modeling (Schriefl et al. 2012). Imaging was conducted on fresh specimens, both before and immediately following the radial tensile tests. Two-photon microscopy imaging (SHG/TPEF) was performed in two separate, ex-situ phases: immediately before the mechanical protocol (baseline configuration) and approximately 5–10 min post-failure. Following macroscopic rupture and tissue delamination, the separated halves of the specimens were unmounted from the tensile grips and transferred to the microscope objective. To account for the impossibility of tracking identical micrometric coordinates pre- and post-test, and to mitigate the influence of elastic recoil and viscoelastic relaxation, a statistical sampling strategy was adopted. For each specimen, 5 distinct regions of interest (ROIs) were imaged across multiple transmural depths at baseline, and 5 distinct ROIs were imaged post-failure (totaling 50 3D image stacks across the cohort). The structural fiber orientation angles and the dispersion parameter κ were processed and averaged across these multiple spatial domains, providing a homogenized representation of the permanent microstructural damage and irreversible reorganization induced by radial failure. This approach allowed for the identification of the initial fiber orientation and dispersion, which serve as key input parameters for the solid phase of the biphasic model. The microstructural data were specifically used to inform the viscoelastic-damage framework by identifying the density and alignment of the lamellar fibers. By comparing pre and post-test images, the extent of microstructural degradation (e.g., collagen fiber straightening or elastin bundle rupture) was qualitatively assessed to justify the damage evolution laws implemented in the computational model. This ensures that the simulated damage propagation reflects the actual physical changes observed at the microscale.

### Constitutive model: reactive viscoelastic damage

To capture the complex interaction between fluid redistribution and tissue degradation, the solid phase of the aortic wall is governed by a reactive viscoelastic damage model. As outlined in the methodological workflow (Fig. 1), this constitutive theory provides the mathematical basis for both the analytical parameter identification and the finite element validation. Within this framework, the tissue is conceptualized as a poroelastic medium where the macroscopic response emerges from the coupling between the interstitial fluid dynamics and the intrinsic mechanics of the solid phase (Nims and Ateshian 2017). Although the biphasic nature accounts for fluid-driven dissipation via Darcy’s law, the core of the constitutive development is focused on the solid matrix response. By isolating the constitutive relations of the solid-phase, we can explicitly define how the kinetics of the molecular bonds and the evolution of damage regulate stress $\boldsymbol{\sigma }_s$, independently of the transient pressures generated by the fluid phase.

#### Reactive viscoelasticity framework

Focusing on the constitutive development of the solid phase, $\boldsymbol{\sigma }_r^s$ is governed by a reactive viscoelastic framework. This approach idealizes the structural matrix as a continuous mixture of multiple molecular networks: a permanent "strong" network providing long-term elastic stability, and multiple generations of "weak" transient networks that undergo continuous dissociation and reformation (Ateshian 2015). In this framework, the total stress of the intact material is the additive sum of the equilibrium elastic response from strong bonds $\boldsymbol{\sigma }_e$ and the time-dependent contribution of multiple generations of weak bonds $\boldsymbol{\sigma }_b$:

1 $$\begin{aligned} \boldsymbol{\sigma }_{r}^{s}(t) = \boldsymbol{\sigma }_{e}(\textbf{F}(t)) + \sum _{u} w_{u} \boldsymbol{\sigma }_{b} (\textbf{F}^{u}(t)) \end{aligned}$$

where $w_u$ represents the rate of bond formation/reforming at time *u*, and $\textbf{F}^u(t)$ is the relative deformation gradient. The weighting factor $w_u$ is governed by a reduced relaxation function $g(t) = e^{-t/\tau }$, where τ represents the characteristic relaxation time of the tissue. This allows the model to capture the intrinsic dissipation of the extracellular matrix independently of fluid flow (Ateshian et al. 2022).

A key feature of the reactive framework is the distinction between the deformation perceived by each bond type. Strong bonds are permanent and respond to the total deformation gradient $\textbf{F}(t)$ relative to the initial state (t=0). In contrast, weak bonds are assumed to be born in a stress-free configuration at their time of formation *u*. Their mechanical response at current time *t* depends solely on the relative deformation gradient:

2 $$\begin{aligned} \textbf{F}^{u}(t) = \textbf{F}(t) \textbf{F}^{-1}(u) \end{aligned}$$

Under the uniaxial strain conditions of the radial tensile test, the deformation gradient simplifies to $\textbf{F}(t) = \text {diag}(1, 1, \lambda _z(t))$. Consequently, the relative stretch $\lambda _{rel}$ for a bond generation formed at time *u* is defined as:

3 $$\begin{aligned} \lambda _{rel}(t, u) = \frac{\lambda _z(t)}{\lambda _z(u)} \end{aligned}$$

This relative stretch is fundamental for capturing stress relaxation: during a period of constant total stretch ($\lambda _z(t) = \text {const}$), newly reformed bonds emerge with $\lambda _{rel} = 1$, thus contributing no stress and leading to the macroscopic decay of the load.

In this study, the permanent strong bonds are modeled using a Neo-Hookean material to represent the isotropic elastic response of elastin-rich matrix. The transient weak bonds are modeled using the Holzapfel-Gasser-Ogden formulation, capturing the anisotropic and non-linear recruitment of collagen fibers, which are considered the primary drivers of tissue’s viscoelastic dissipation.

***Strong bonds–Neo-Hookean***

The permanent solid matrix is modeled as a compressible Neo-Hookean hyperelastic material. In the context of a biphasic framework, the total volume change of the mixture is associated with the deformation of the solid skeleton and the subsequent change in porosity. The strain energy density function $\Psi ^e$ is defined as follows, consistent with the implementation in FEBio:

4 $$\begin{aligned} \Psi ^{e} = \frac{\mu _{e}}{2}(I_{1} - 3) - \mu _{e} \ln J + \frac{\lambda _{e}}{2}(\ln J)^{2} \end{aligned}$$

where $\mu _e$ and $\lambda _{e}$ are the Lamé parameters, related to the Young’s Modulus E, and Poisson’s ratio ν by:

5 $$\begin{aligned} \mu _{e} = \frac{E}{2(1 + \nu )} \quad ; \quad \lambda _{e} = \frac{\nu E}{(1 + \nu )(1 - 2\nu )} \end{aligned}$$

$I_1$ is the first invariant of the right Cauchy-Green deformation tensor $\textbf{C}$ and *J* is the determinant of the deformation gradient $\textbf{F}(t)$, representing the volume ratio (Bonet and Wood 2008; Capaldi 2012). The explicit form of the stress tensor, considering the uniaxial tensile test and the specific boundary conditions imposed, is:

6 $$\begin{aligned} \boldsymbol{\sigma }_{e} = \frac{\mu _{e}}{J} (\textbf{B} - \textbf{I}) + \frac{\lambda _{e}}{J} (\ln J) \textbf{I} \end{aligned}$$

By extracting the radial component $\sigma _{zz}^e$ and substituting the kinematic relations $J = \lambda _z(t)$ and $B_{zz} = \lambda _z^2(t)$, the final expression for the radial stress is:

7 $$\begin{aligned} \sigma _{e,zz} (\lambda _{z}(t)) = \underbrace{\frac{\mu _{e}}{\lambda _{z}(t)} \left( \lambda _{z}^{2}(t) - 1\right) }_{\text {Distortional response}} + \underbrace{\frac{\lambda _{e}}{\lambda _{z}(t)} \ln (\lambda _{z}(t))}_{\text {Volumetric response}} \end{aligned}$$

The derived formulation highlights the dual mechanical response of the solid matrix under radial loading. The first term represents the isochoric (distortional) elastic response, capturing the resistance of the extracellular matrix fibers to changes in shape. The second term accounts for the volumetric response; since the solid phase is intrinsically incompressible, any change in *J* corresponds to pore dilation. Therefore, $\lambda _{e}$ governs the energetic cost associated with this volume increase and the subsequent interstitial fluid redistribution. For $\lambda _z> 1$, both terms contribute positively to the total stress, reflecting the macroscopic stiffening of the aortic tissue during expansion.

***Weak bonds–Holzapfel-Gasser-Ogden*** The transient weak bonds are described by the Holzapfel-Gasser-Ogden (HGO) unconstrained formulation (Holzapfel 2006; Gasser et al. 2006). Unlike the perfectly incompressible case, this model takes into account the volumetric variations associated with pore dilation in the biphasic framework, consistent with the implementation of unconstrained anisotropic materials in the FEBio software (Maas et al. 2012). The strain energy density $\Psi _r^b$ for a bond generation formed at time *u* is given by:

8 $$\begin{aligned} \Psi _{r}^{b} = \Psi _{\textrm{matrix}}^{b} + \Psi _{\textrm{fibers}}^{b} + \Psi _{\textrm{vol}}^{b} \end{aligned}$$

Under the prescribed uniaxial strain condition, the relative deformation gradient for a generation born at time *u* is $\textbf{F}^u =\mathrm{diag} (1, 1, \lambda _{rel}(t,u))$. Consistent with the FEBio implementation for HGO unconstrained materials, the individual energy terms are defined as:

9 $$\begin{aligned} \Psi _{\textrm{matrix}}^{b} = \frac{c}{2}(I_{1} - 3 - 2 \ln J_{u}) \end{aligned}$$

10 $$\begin{aligned} \Psi _{\textrm{fibers}}^{b} = \frac{k_{1}}{2k_{2}} \sum _{\alpha =1}^{2} \left( \exp \left( k_{2} \langle E_{\alpha } \rangle ^{2} \right) - 1 \right) \end{aligned}$$

11 $$\begin{aligned} \Psi _{\textrm{vol}}^{b} = \frac{K}{2} \left( \frac{J_{u}^{2} - 1}{2} - \ln J_{u} \right) \end{aligned}$$

where *c* is the matrix shear modulus, $k_1, k_2$ are fiber stiffness parameters, and *K* is the bulk modulus. The fiber strain $E_\alpha$ accounts for the structural dispersion κ:

12 $$\begin{aligned} E_{\alpha } = \kappa (I_{1} - 3) + (1 - 3\kappa )(I_{4\alpha } - 1) \end{aligned}$$

where $I_{4\alpha } = \textbf{a}_\alpha \cdot \textbf{C}_u \cdot \textbf{a}_\alpha = \cos ^2\theta + \lambda _{rel}^2 \sin ^2\theta$ and θ represents the angle between the two families of symmetric fibers, which in this model lie on the plane perpendicular to the loading axis *z*.

The radial Cauchy stress $\sigma _{zz}^{b}$ is obtained by differentiating the energy potential with respect to the relative stretch. The final expression for the radial stress component of the HGO weak bonds is:

13 $$\begin{aligned} \begin{aligned} \sigma _{{b,zz}} \left( {\lambda _{{{\text {rel}}}} } \right)&= \frac{{c + \frac{K}{2}}}{{\lambda _{{{\text {rel}}}} }}\left( {\lambda _{{{\text {rel}}}}^{2} - 1} \right) \\&\quad + 4S_{{{\text {fib}}}} \left( {\lambda _{{{\text {rel}}}} } \right) \left[ {\kappa \lambda _{{{\text {rel}}}} + \left( {1 - 3\kappa } \right) \lambda _{{{\text {rel}}}} \sin ^{2} \theta } \right] \\ \end{aligned} \end{aligned}$$

where $S_\text {fib}$ is the auxiliary function for the fiber contribution to the Cauchy stress:

14 $$\begin{aligned} S_{\textrm{fib}}(\lambda _{\textrm{rel}}) = k_{1} \langle E_{\alpha }(\lambda _{\textrm{rel}}) \rangle \exp \left[ k_{2} \langle E_{\alpha }(\lambda _{\textrm{rel}}) \rangle ^{2} \right] \end{aligned}$$

This formulation allows the model to capture the non-linear recruitment of collagen fibers during radial expansion. The complete numerical framework for the identification of these constitutive laws is reported in B

In the computational implementation, the continuous process of bond reforming is discretized into *n* time steps. The total radial stress of the intact solid matrix $\sigma _{r,zz}^s(t)$ (from 1) is calculated as the sum of the equilibrium response from the permanent strong bonds and the weighted contributions of all weak bond generations formed up to the current time *t*:

15 $$\begin{aligned} \sigma _{r,zz}^{s}(t) = \sigma _{e,zz} (\lambda _{z}(t)) + \sum _{i=1}^{n} w_{i}(t) \, \sigma _{b,zz}(\lambda _{\textrm{rel}}(t, u_{i})) \end{aligned}$$

where the weighting factor $w_i(t)$ represents the fraction of bonds formed at time $u_i$ that have survived until time *t*, governed by the relaxation function $g(t-u_i) = e^{-(t-u_i)/\tau }$. During the 30-second relaxation phase, the total stretch $\lambda _z$ remains constant. As time progresses, new generations of bonds form with a relative stretching $\lambda _\text {rel} = 1$. Since newly formed generations emerge in the current configuration with zero initial stress $\sigma _{zz}^{b}(t) = 0$, they do not immediately contribute to the load, whereas the weight of older, strained generations decays exponentially over time. This summation mechanism effectively captures the macroscopic stress relaxation observed in radial tensile tests.

#### Damage evolution

To account for the progressive failure of the inter-lamellar microstructure, a damage variable *D* is introduced. The total Cauchy stress of the solid phase $\sigma _{zz}^s$ is obtained by:

16 $$\begin{aligned} \sigma _{zz}^{s}(t) = (1 - D(t)) \, \sigma _{r,zz}^{s}(t) \end{aligned}$$

where $\sigma ^s_{r,zz}(t)$ represents the stress state of the intact reactive viscoelastic matrix. Damage initiation and evolution are governed by a scalar driving quantity, $\Xi _m(t)$. To guarantee path-dependency and the irreversibility of the degradation process, $\Xi _m(t)$ tracks the historical maximum achieved by the strain energy density function of the intact material ($\Psi _{\text {0}}$) over the loading history up to the current time *t*:

17 $$\begin{aligned} \Xi _m(t) = \max _{-\infty < s \le t} \Psi _0\big (\textbf{C}_u(s)\big ) \end{aligned}$$

Here, $\Psi _{\text {0}}$ accounts for the coupled energetic contribution of both the isotropic ground substance and the anisotropic fiber families. The damage variable *D*(*t*) follows a log-normal cumulative distribution function, which provides a smooth transition from intact to degraded states. This probabilistic formulation is particularly effective for capturing the stochastic nature of microstructural fiber rupture in biological tissues (Ateshian et al. 2022). Within the FEBio framework, this implementation allows for a robust stabilization of the numerical softening:

18 $$\begin{aligned} D(t) = D_\text {max} \cdot \frac{1}{2} \operatorname {erfc} \left[ -\frac{\ln (\Xi _{m}(t)/\mu )}{\sigma \sqrt{2}} \right] \end{aligned}$$

where μ and σ are the location and spread parameters, respectively, which control the sensitivity of the inter-lamellar bonds to deformation. As the radial expansion increases and exceeds the damage threshold, *D* evolves from 0 to its maximum value $D_\text {max}$, representing the irreversible loss of mechanical integrity and the onset of tissue delamination observed during experimental radial tensile tests.

To prevent over-parameterization and avoid numerical uniqueness issues stemming from parameter cross-correlations, a two-step calibration protocol was adopted. Initial screening on a subset of experimental curves established the characteristic interlamellar damage threshold of the tissue, which consistently centered around a critical strain energy density of μ=3.50 kPa with an accumulation profile described by σ=0.60. In the second phase, these two microstructural priors were locked as constant traits across the entire cohort. This isolated $D_{\max }$ as the sole specimen-specific free parameter, ensuring a unique and rigorous quantification of localized tissue degradation (Table 2).

Physically, while the log-normal cumulative framework implies that damage initiation is mathematically continuous from the onset of loading without a sharp deterministic threshold, the variable *D* remains strictly negligible within the physiological strain range. This continuous configuration represents the stochastic nature of biological soft tissues, where microstructural heterogeneity dictates that a baseline, sub-clinical fraction of sacrificial molecular links may fail early under localized stress concentrations. From a computational standpoint, a smooth formulation is highly preferred over a sharp, discontinuous threshold. Avoiding sudden jumps in material stiffness allows the FEBio solver to maintain numerical stability and converge smoothly during tissue degradation. The complete thermodynamic derivation, the explicit mathematical tensor projections, and the physical meaning of the driving invariants are detailed in Appendix C.

Equation 16 underscores the multi-physics nature of the model, as the radial load $\sigma _{zz}(t)$ encapsulates the interaction of several distinct mechanical phenomena, each governed by specific material parameters:

1. **Matrix elasticity** Young’s Modulus E, and Poisson’s ratio ν of the Neo-Hookean phase;
2. **Anisotropic reinforcement** the HGO fiber parameters $c, k_1, k_2, \kappa , \gamma$, which capture the non-linear recruitment and orientation of collagen;
3. **Intrinsic dissipation** the characteristic relaxation time τ, which governs the bond-reforming kinetics via the weighting factors $w_i(t)$;
4. **Progressive failure** the damage variable *D*(*t*), which tracks the history-dependent loss of inter-lamellar cohesion.

By integrating these components, the proposed framework provides a robust tool to model the intrinsic molecular relaxation with bond reforming. Most importantly, it allows for a quantitative representation of the progressive softening that precedes total aortic dissection, mapping macroscopic experimental data to internal tissue degradation. For the full derivation of constitutive relations, the reader is referred to Appendix B.

### Analytical calibration of solid matrix parameters

The constitutive framework described in Sect. 2.6 represents the theoretical physics governing the aortic solid matrix. To identify the material parameters from experimental data, the research strategy relies on an analytical model as the primary calibration tool, as illustrated in the methodological workflow (Fig. 1). The rationale behind this choice is to decouple the intrinsic material properties from the secondary structural effects. By assuming a localized uniaxial radial strain state, the complex constitutive equations are resolved into a closed-form analytical expression implemented in Wolfram Mathematica. This approach offers significant advantages for parameter identification; specifically, it provides a robust and high-speed environment for non-linear least-squares optimization, allowing for the simultaneous fitting of multiple loading-relaxation cycles across all specimens. To guarantee numerical stability and address the non-convex nature of the parameter space, a rigorous multi-start optimization protocol was embedded directly into this analytical phase. For each specimen, the non-linear least-squares optimization loop was repeated automatically using multiple initial parameter guesses, which were randomly generated across three orders of magnitude within physically acceptable boundaries. Furthermore, by isolating the solid-phase mechanics, the analytical model ensures that the identified stiffness ($E, c, k_1, k_2$), viscoelastic (τ), and damage ($D_\text {max}$) parameters specifically represent the molecular bond kinetics and interlamellar integrity. This strategy minimizes the risk of numerical artifacts that could otherwise arise from a direct full-scale finite element optimization, ensuring the physical identifiability of the constitutive properties. Indeed, the integration of experimental structural information or strain data has been shown to be essential for the unique and robust identification of the anisotropic properties of the aortic wall (Avril et al. 2010). This analytical stage represents the calibration phase of our workflow, where the intrinsic properties of the aortic media are quantified. To verify that these identified parameters remain consistent within a multi-physics environment, they are subsequently integrated into a comprehensive finite element framework, as described in the following section.

### Finite element model for poromechanical validation

To numerically investigate the damage mechanisms and the fluid-solid coupling, a computational model was developed in FEBio. While the analytical approach focuses on the intrinsic solid matrix, the FE model explicitly accounts for the poroelastic nature of the tissue. The framework is designed to replicate the specific mechanical environment of the radial tensile test, providing a bridge between macroscopic observations and internal tissue degradation. In fact, a zero displacement condition is applied to the lower surface of the sample in all 3 directions, to represent the fixed plate of the traction machine. In contrast, active displacement was applied to the top surface; here, the radial displacement $d_z(t)$ was imposed as a piecewise function using a Heaviside step formulation:

19 $$\begin{aligned} d_{z}(t) = \sum _{n=1}^{N} \Delta (d_{z})_{n} \cdot H(t - t_{n-1}) \end{aligned}$$

where *N* is the total number of loading increments, $\Delta (d_z)_n \approx 0.082$ mm is the displacement applied at each step, and H(·) is the Heaviside function. This discrete approach ensures that each deformation step and the subsequent 30-second relaxation period are captured with the same temporal fidelity as the experimental protocol, allowing the poromechanical and viscoelastic effects to evolve naturally within the simulation.

Regarding fluid transport, a zero fluid flux condition was enforced on both the top and bottom surfaces, representing the impermeable interface between the tissue and the metal platens. This configuration forces the interstitial fluid to redistribute internally or exit through the lateral boundaries during the tensile event, capturing the coupled poromechanical response of the wall. Similar boundary conditions have been successfully employed to model the indentation and traction of hydrated soft tissues in FEBio, ensuring a realistic representation of the fluid-solid interaction under confined or semi-confined loading (Smoljkić et al. 2016).

The constitutive framework was specifically designed to integrate the three main phenomena observed in the aortic media under radial tension. First, a biphasic formulation accounts for the interaction between the solid extracellular matrix and the interstitial fluid, capturing the pressure-driven effects. This is coupled with a reactive viscoelastic framework that describes the intrinsic time-dependent response of the solid phase during the relaxation period. Finally, a dedicated damage function was integrated to simulate the progressive degradation of mechanical properties, effectively representing the failure of interlamellar cohesion that precedes total aortic dissection.

### Parameter identification and numerical implementation

The quantitative identification of the material parameters was performed through a sequential optimization procedure. As a first step, the analytical model (Sect. 2.7) was used to provide a stable initialization by fitting individual loading-relaxation cycles. This preliminary estimation allowed for a robust calibration of the stiffness ($E,\nu , c, k_1, k_2$), relaxation (τ), and damage ($D_\text {max}$) parameters against the experimental stress-time data.

Subsequently, the model’s accuracy was further refined within the FEBio environment to ensure consistency with the coupled poromechanical response (Sect. 2.8). The optimization aimed to minimize the normalized residual *r* between the predicted Cauchy stress ($\sigma _\text {model}$) and the experimental data ($\sigma _\text {exp}$):

20 $$\begin{aligned} r(t) = \frac{\sigma _{\text {model}}(t) - \sigma _{\text {exp}}(t)}{\max \left( | \sigma _{\text {exp}}(t) | \right) } \end{aligned}$$

The minimization of the normalized residual *r*(*t*) was evaluated against localized parameter perturbations to ensure that the final calibrated values represent a stable global minimum rather than an artifact of local convergence.

This inverse identification pipeline was applied independently to each of the five selected experimental curves. The representative constitutive properties of the porcine descending aorta were then established by evaluating the cohort-wide arithmetic mean and standard deviation of the parameters extracted from the individual optimization runs. The precision of the final fit was quantitatively assessed using the coefficient of determination ($R^2$) and the root mean square error (RMSE):

21 $$\begin{aligned} R^{2} = 1 - \frac{\sum _{i=1}^{m} (\sigma _{\textrm{exp}}(t_{i}) - \sigma _{\textrm{model}}(t_{i}))^{2}}{\sum _{i=1}^{m} (\sigma _{\textrm{exp}}(t_{i}) - \bar{\sigma }_{\textrm{exp}})^{2}}\end{aligned}$$

22 $$\begin{aligned} \textrm{RMSE} = \sqrt{\frac{1}{m} \sum _{i=1}^{m} (\sigma _{\textrm{exp}}(t_{i}) - \sigma _{\textrm{model}}(t_{i}))^{2}} \end{aligned}$$

where *m* is the number of data points per test. High $R^2$ values (>0.95) and low RMSE were considered requirements for a successful fit, ensuring the model effectively captures both peak stresses and relaxation kinetics.

## Results

### Mechanical response of aortic tissue under radial tensile loading

**Fig. 3:**
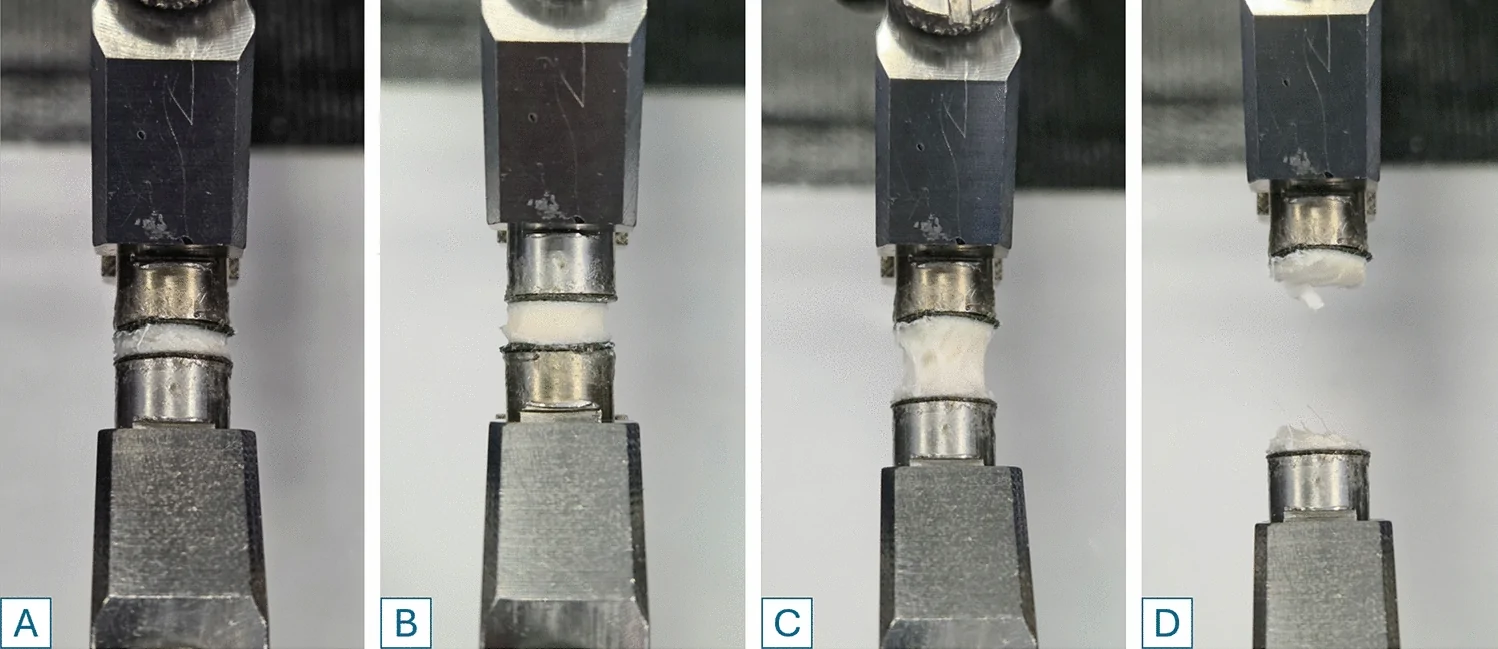
Representative stages of the step-relaxation radial tensile test: **A** initial configuration after the first increment of 0.082 mm; **B** intermediate state (approx. step 25) showing substantial elongation; **C** pre-failure state characterized by maximum stretch, thinning, and onset of interlamellar delamination; **D** final mechanical rupture of the specimen

The incremental, step-relaxation radial tensile test was successfully performed on the total pool of twenty cylindrical aortic samples harvested across the cohort. Following the single-animal representative selection criteria detailed in Sect. 2.4, Fig. 3 illustrates the morphological evolution of a typical specimen throughout the loading protocol. All samples exhibited remarkable hyperelastic deformability, sustaining a radial stretch exceeding λ=2 before reaching ultimate mechanical failure. The progression of the test is characterized by different phases: initially, the tissue undergoes a small-strain regime (Fig. 3A) where the first 0.082 mm step triggers the poromechanical response described previously. As the displacement increments accumulate—reaching approximately step 25 (Fig. 3B)—the specimen undergoes substantial macroscopic deformation, doubling its initial length. Approaching the final stages of the test (Figure 3C), the material enters a high-strain regime where the interlamellar structure reaches its limit of extensibility. Finally, ultimate mechanical failure occurs (Fig. 3D), marked by a sudden drop in the recorded load and the complete structural rupture of the sample.

**Fig. 4:**
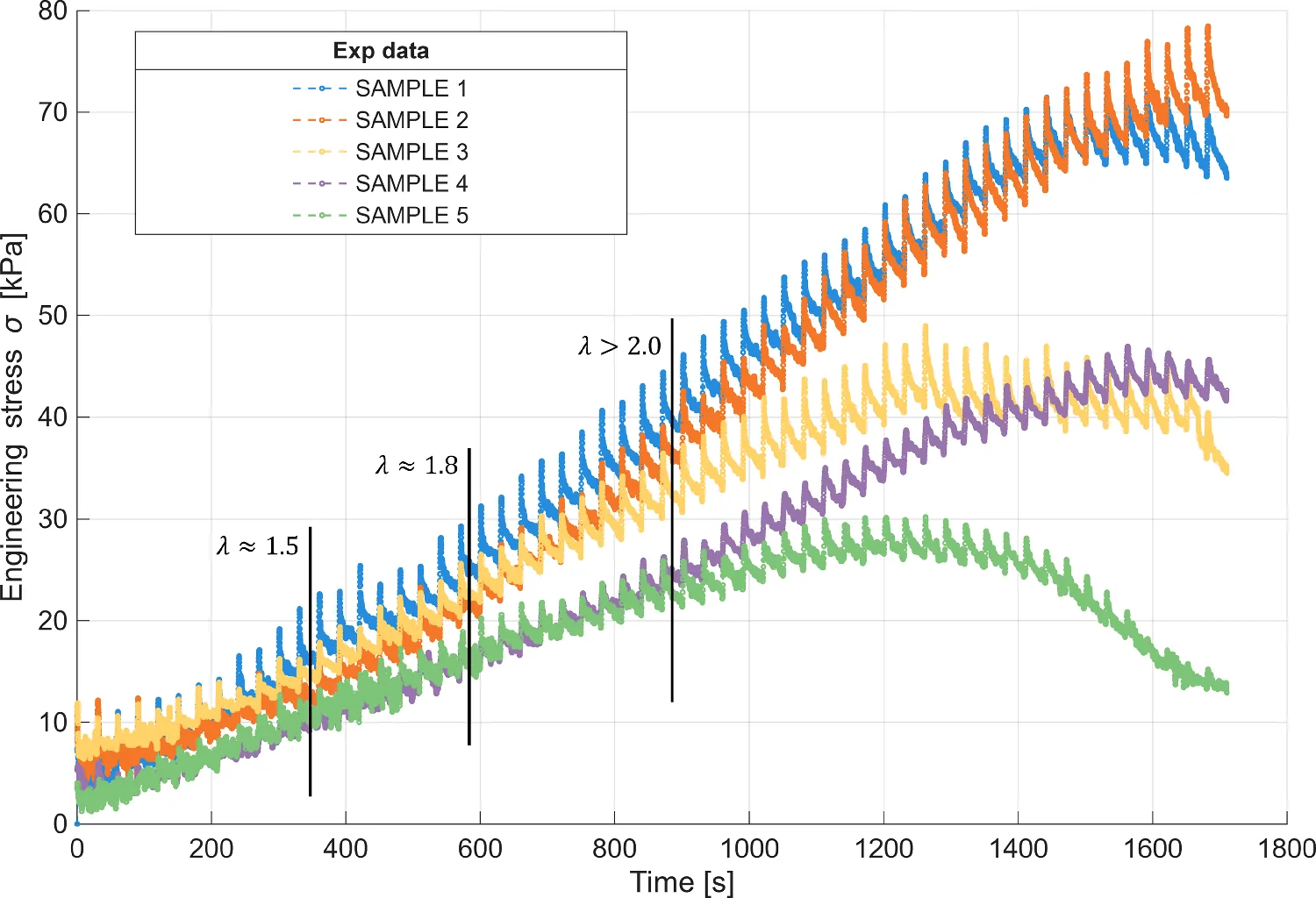
Experimental engineering stress–time curves of the aortic specimens under multi-step radial tension. The plot is enhanced with strategic kinematic indicators, highlighted by vertical dashed lines at key deformation thresholds calculated from the 2.0 mm initial tissue thickness: $\lambda \approx 1.5$ (representing the moderate out-of-plane strain threshold at the 12th step), $\lambda \approx 1.8$ (capturing the microstructural damage acceleration regime at the 20th step), and λ>2.0 (identifying the deep damage state at the 25th step preceding ultimate macroscopic failure)

The experimental engineering stress–time curves for the five analyzed specimens are presented in Fig. 4. Due to the small radial dimensions and resulting low force magnitudes, raw data were processed using a Savitzky–Golay filter to mitigate experimental noise, preserving the underlying mechanical trends. The results demonstrate a consistent, non-linear mechanical response across all samples. As the displacement increments accumulate—reaching a stretch of $\lambda \approx 1.5$ (indicated by the moderate-strain pointer in Fig. 4)—the specimen undergoes substantial macroscopic deformation. At higher stretch levels ($\lambda \approx 1.5-1.8$, represented by the shaded damage-initiation band in Fig. 4), a distinct alteration in the stress profile is observed, appearing as a subtle plateau and a marked change in curvature. This phase corresponds to the progressive exhaustion of lamellar extensibility. Finally, ultimate mechanical failure occurs (λ>2.0, marked by the ultimate failure indicator in Fig. 4), characterized by a sudden drop in the recorded load and the complete structural rupture of the sample. This multi-stage process demonstrates the tissue’s ability to redistribute internal stresses through both fluid motion and matrix viscoelasticity before irreversible damage leads to failure.

Analysis of the experimental response reveals three distinctive mechanical features of the aortic tissue under radial loading. First, a pronounced stepwise viscoelastic decay towards a relaxed plateau is observed during the 30-second hold following each instantaneous stress peak, indicating a strong coupled contribution of solid-matrix viscoelasticity and fluid poromechanics. Second, a marked non-linear radial stiffening effect becomes visible as the loading protocol progresses, manifested by a cumulative increase in the peak stress amplitude. Notably, the stress magnitudes recorded in the radial direction remain significantly lower (on the order of kPa) than those typically reported for axial or circumferential loading (on the order of MPa), which confirms the radial axis as the most compliant and mechanically vulnerable direction of the vessel (Holzapfel 2017). Finally, a clear progressive damage accumulation and subsequent loss of structural integrity can be identified in the high-strain region (1.5<λ<1.8), where the subtle plateau and characteristic change in curvature precede total failure. This overall mechanical behavior is consistent with previous observations reported for arterial tissues subjected to multi-step relaxation protocols (Gültekin et al. 2019; Valente et al. 2024; Leng et al. 2018). Despite a cumulative radial stretch exceeding 200% across the full multi-step protocol, the assumption of a small-strain incremental window is physically motivated by the transient poromechanical and fiber-reinforcement constraints acting at the onset of each relaxation phase, as detailed in Sect. 2.4.

### Microstructural reorganization induced by radial tension

**Fig. 5:**
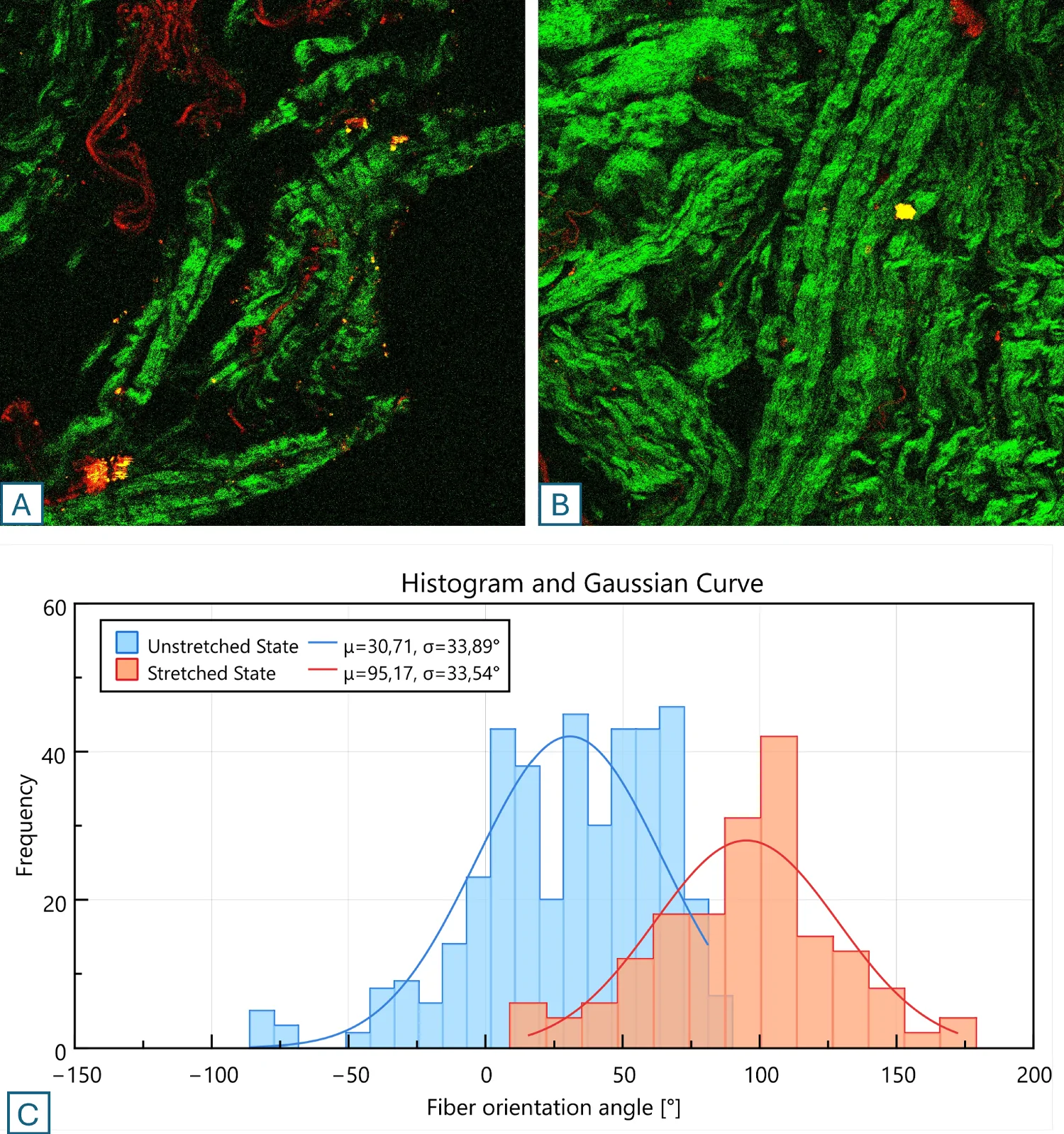
Microstructural reorganization of the aortic media. Merged SHG (collagen, green) and TPEF (elastin, red) image in the unstretched state (**A**) and fully stretched state (**B**); Gaussian distribution of fiber orientation from $\mu \approx 31^\circ$ in unstretched state to $\mu \approx 95^\circ$ in stretched state (**C**)

To provide a structural basis for the observed mechanical response, two-photon microscopy (SHG/TPEF) was utilized to visualize the collagen and elastin networks (Fig. 5). The comparison between the unstretched (Fig. 5A) and fully stretched states (Fig. 5B) reveals a profound microstructural reorganization within the tangential plane of the aortic media. The quantitative analysis of collagen fiber orientation documents a significant angular shift induced by the radial load. In the unstretched state, fibers exhibit an initial mean orientation of approximately $31^\circ$ (Fig. 5C), which rotates toward a fully aligned state of approximately $95^\circ$ under maximum radial stretch (Fig. 5C).

Based on these microstructural observations, the local material axes in FEBio were oriented to align the local $e_1,e_2$ plane with the tangential plane of the aortic media. The mean in-plane fiber angle γ was set to a fixed prior of $60 ^\circ$ to serve as a representative mechanical average of the planar collagen network architecture. Maintaining $\gamma =60^\circ$ and the structural dispersion κ=0.10 as fixed priors decouples the geometric layout from the material stiffnesses ($k_1, k_2$), preventing numerical parameter cross-correlation and ensuring unique parameter identification.

### Identification and coherence of constitutive parameters

**Fig. 6:**
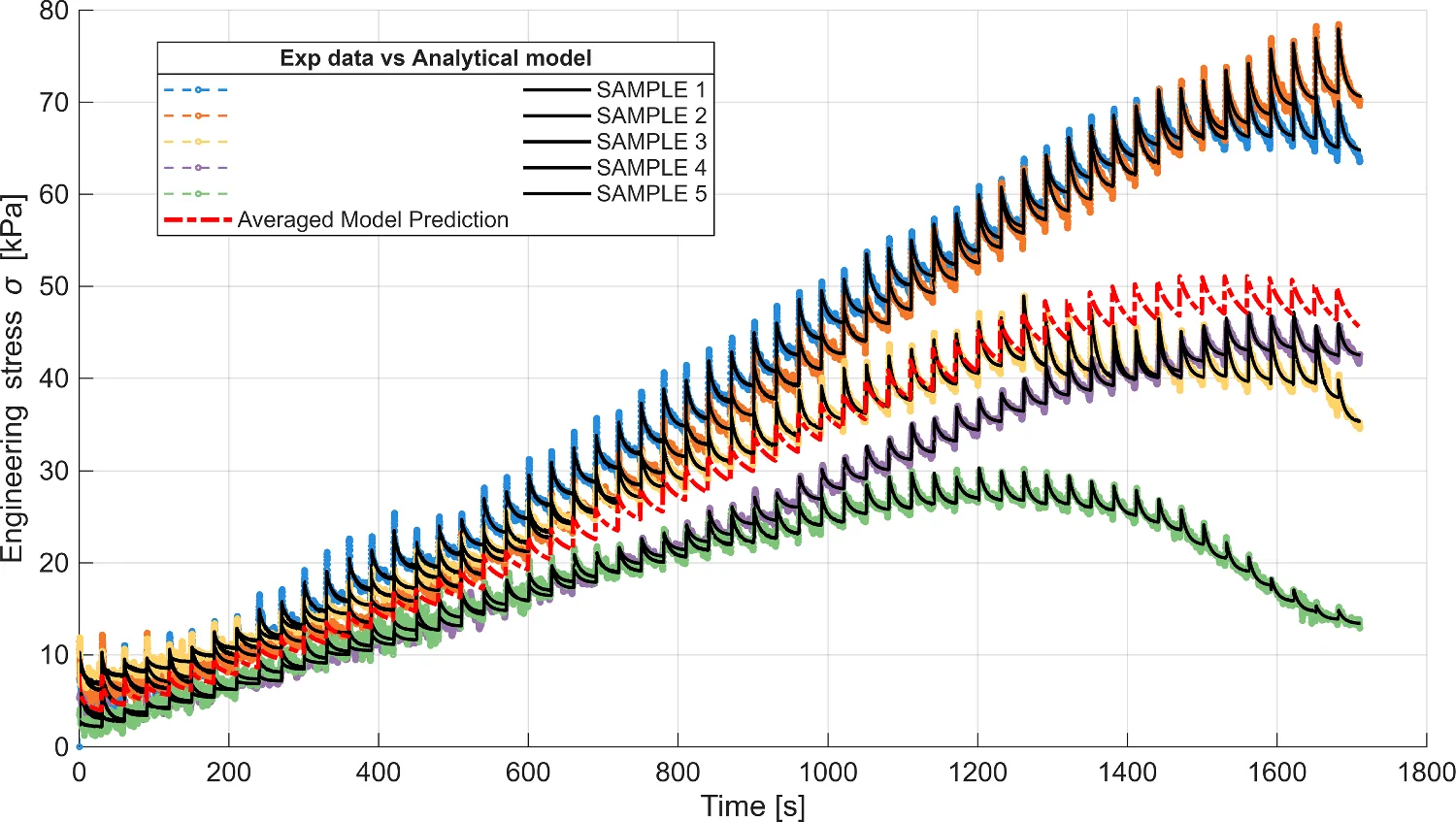
Comparison between experimental engineering stress–time curves (dashed colored lines) and the individual model predictions (thin solid black lines) for the analyzed specimens (n=5). The thick red dashed line represents the global ensemble average of the model predictions, demonstrating the capacity of the constitutive framework to capture the central trend of the mechanical envelope without non-linear parameter distortion

The inverse parameter identification was performed by fitting the individual experimental stress–time curves (n=5) to the analytical expression of the solid phase within the reactive-damage framework. During this stage, the material is treated as an intrinsically incompressible solid matrix ($\nu \approx 0.5$). This choice is physically justified by the high water content of the aortic media, which prevents significant volume changes of the solid phase during rapid loading phases. As shown in Fig. 6, the calibrated reactive viscoelastic-damage model successfully captures the experimental radial mechanical response across all tested samples. To evaluate the global performance of the framework without introducing non-linear numerical distortions, a representative model response was generated via an ensemble average by computing the point-by-point mean of the five individual model predictions.

The resulting parameters, representing the elastic matrix, fiber recruitment, and progressive damage, are summarized in Table 2. A defining result of this study is the magnitude of the identified stiffness parameters. The elastic moduli, *E* and *c* and the fiber stiffness $k_1$ are in the range of 2.5–10.5 kPa. These values are approximately two to three orders of magnitude lower than those typically reported for the circumferential and axial directions of the porcine aorta, which usually range from 0.1 to 1.5 MPa (Holzapfel et al. 2002; Gasser and Holzapfel 2006). This striking discrepancy is physically meaningful: while in-plane tests primarily probe the high-stiffness collagen networks, the radial tensile test targets the interlamellar ground matrix and the relatively weak cross-links between elastic lamellae. The low values identified here represent the mechanical weak link of the aortic wall, providing a quantitative basis for its susceptibility to delamination during dissection.

**Table 2:** Material parameters and microstructural priors, and damage law thresholds identified for the aortic tissue model

**Parameter**	**Value (Mean ± Std. Dev.)**
*E* (Young’s modulus)	10.525 ± 0.008 kPa
ν (Poisson’s ratio)	0.49 ± 0.15
*c* (Matrix shear)	2.52 ± 0.09 kPa
$k_1$ (Fiber stiffness)	8.04 ± 0.83 kPa
$k_2$ (Fiber non-linearity)	1.810 ± 0.001
τ (Relaxation time)	8.00 ± 4.43 s
$D_{\max }$ (Maximum allowable damage)	0.60 ± 0.07
**Fixed microstructural and damage priors**	
γ (Fiber angle)	60$^\circ$
κ (Dispersion)	0.10
μ (Damage energy location parameter)	3.50 kPa
σ (Damage accumulation spread)	0.60

Values are reported as mean ± standard deviation calculated across the independent dataset of the five selected specimen calibrations (n=5)

The model effectively achieves a clear separation of mechanical contributions. The Neo-Hookean term *E* and matrix shear *c* represent the isotropic contribution of the elastin-rich ground substance, which dominates the initial low-strain regime. The HGO parameters ($k_1, k_2$) capture the non-linear strain-stiffening as collagen fibers reorient toward the transverse plane, as confirmed by the microscopy evidence. The damage parameter $D_\text {max} \approx 0.60$ indicates that the tissue loses approximately 60% of its load-bearing capacity due to microstructural failure before total macroscopic rupture occurs. This highlights that damage is a progressive phenomenon rather than a sudden event. The robustness of the identification procedure is further supported by the numerical uniqueness of the solution. Across all executed multi-start numerical trials, the solver consistently converged to the same global minimum regardless of the initial parameter guess. This insensitivity to the starting points confirms that the optimization landscape is well-behaved and free of problematic local minima. This stability demonstrates that integrating independent microstructural priors ($\gamma = 60^\circ , \kappa = 0.1$) and locking the baseline damage thresholds ($\mu = 3.50\text { kPa}, \sigma = 0.60$) successfully prevented parameter cross-correlation and mutual compensation among the remaining free parameters. Finally, the inter-sample variability observed, particularly for the relaxation time τ (8.0±4.43 s), reflects the inherent heterogeneity of the specimens. This variability is likely linked to differences in local porosity and fluid volume fraction, which govern the poromechanical component of the relaxation. The consistency of the remaining parameters across the five samples confirms that the proposed biphasic-damage framework is a reliable tool for capturing the fundamental radial physics of the aorta.

### Model performance: reproduction of radial tensile response

The core achievement of the proposed framework lies in its ability to simultaneously reproduce three distinct and competing mechanical phenomena within a single constitutive environment: non-linear stiffening, multi-scale time-dependent relaxation, and progressive damage evolution. As illustrated in Fig. 6, the model tracks the full experimental stress–time history with high fidelity, yielding an average $R^2 = 0.93 \pm 0.04$ and a global RMSE of 2.5±0.8 kPa, values comparable with other studies (Ogden et al. 2004). Specifically, the simulation accurately replicates the stress decay during the 30-second dwell periods by coupling Darcy’s law with the reactive viscoelastic framework, thereby distinguishing between rapid, pressure-driven fluid redistribution and the longer-term molecular rearrangement of the solid matrix. Parallel to these temporal effects, the non-linear recruitment of collagen fibers—captured through the HGO formulation-enables the model to follow the characteristic exponential increase in peak stresses as radial stretch exceeds 150%. Furthermore, a critical highlight of the model’s performance is the reproduction of the plateauing effect and the subtle alterations in curvature observed at higher strain levels; in this regime, the damage variable *D* effectively modulates the reactive stress to represent the irreversible loss of interlamellar cohesion. This simultaneous capture of diverse behaviors demonstrates that the model is not merely a mathematical curve-fitting exercise but possesses deep mechanistic consistency. Each parameter corresponds to a specific physical process–fluid flux, fiber alignment, or bond failure–occurring within the medial layer.

The ability to maintain such high statistical accuracy across the entire loading protocol, including the pre-failure regime, positions this framework as a unique tool for quantifying the mechanical precursors of aortic dissection. This simultaneous capture of diverse behaviors demonstrates that the model possesses deep mechanistic consistency, where each parameter corresponds to a specific physical process–such as fluid flux, fiber recruitment, or bond failure–occurring within the medial layer. Such an approach aligns with the histomechanical modeling of porcine vascular tissues, which emphasizes the necessity of multi-fiber formulations to faithfully replicate the physiological and high-pressure response (Sokolis and Sokolis 2012). Furthermore, by bridging microscale interactions with macroscopic mechanical states (Eichinger et al. 2021), the framework ensures that the identified parameters are not merely the result of a mathematical curve-fitting exercise but represent a consistent reflection of the tissue’s underlying constituents.

### Numerical validation of the FEBio implementation

**Fig. 7:**
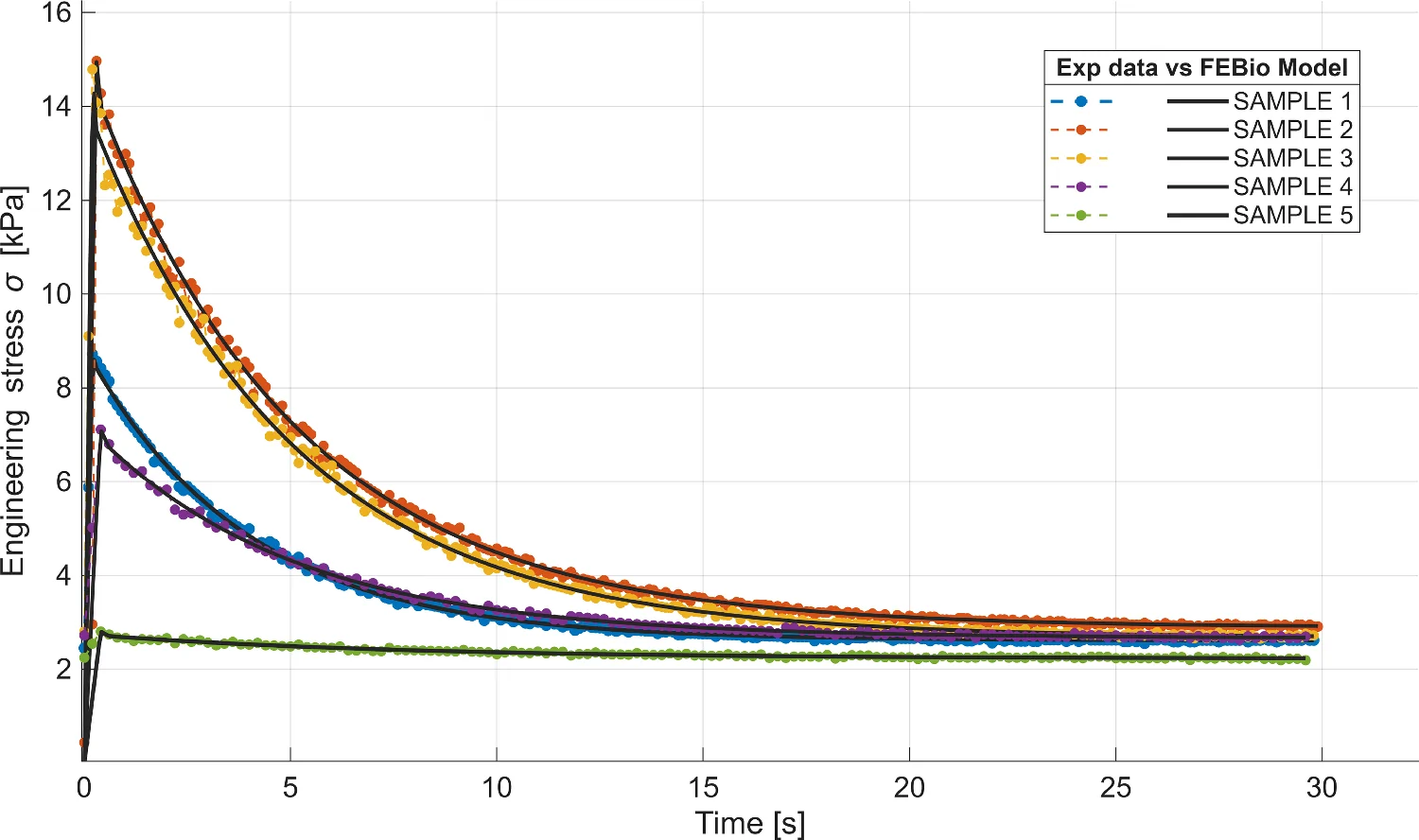
Validation of the numerical framework (first relaxation step). Experimental engineering stress (colored markers) versus FEBio simulations (solid black lines) for the five aortic specimens. The overlap confirms the model’s accuracy in capturing dissipative kinetics and validates the numerical implementation

To validate the numerical implementation and investigate the role of interstitial fluid flow, the experimental engineering stress-time curves were compared with biphasic finite element simulations in FEBio. Fig. 7 show the results of the first relaxation step. Unlike the analytical identification, the numerical model explicitly accounts for the poroelastic nature of the tissue by solving for the interstitial fluid pressure (*p*) and the fluid flux across the specimen boundaries. To solve these coupled poromechanical equations, the boundary conditions of the finite element model were matched to the experimental configuration. The top and bottom surfaces in contact with the rigid platens were prescribed as impermeable (zero fluid flux). Conversely, the lateral free surface of the specimen was prescribed with a constant fluid pressure of p=0MPa to simulate immersion within the unpressurized external PBS bath at atmospheric pressure. This configuration ensures that fluid flow across the lateral boundary is dictated entirely by internal poromechanical gradients, allowing for the uninhibited simulation of the inward suction and outward exudation phases.

**Fig. 8:**
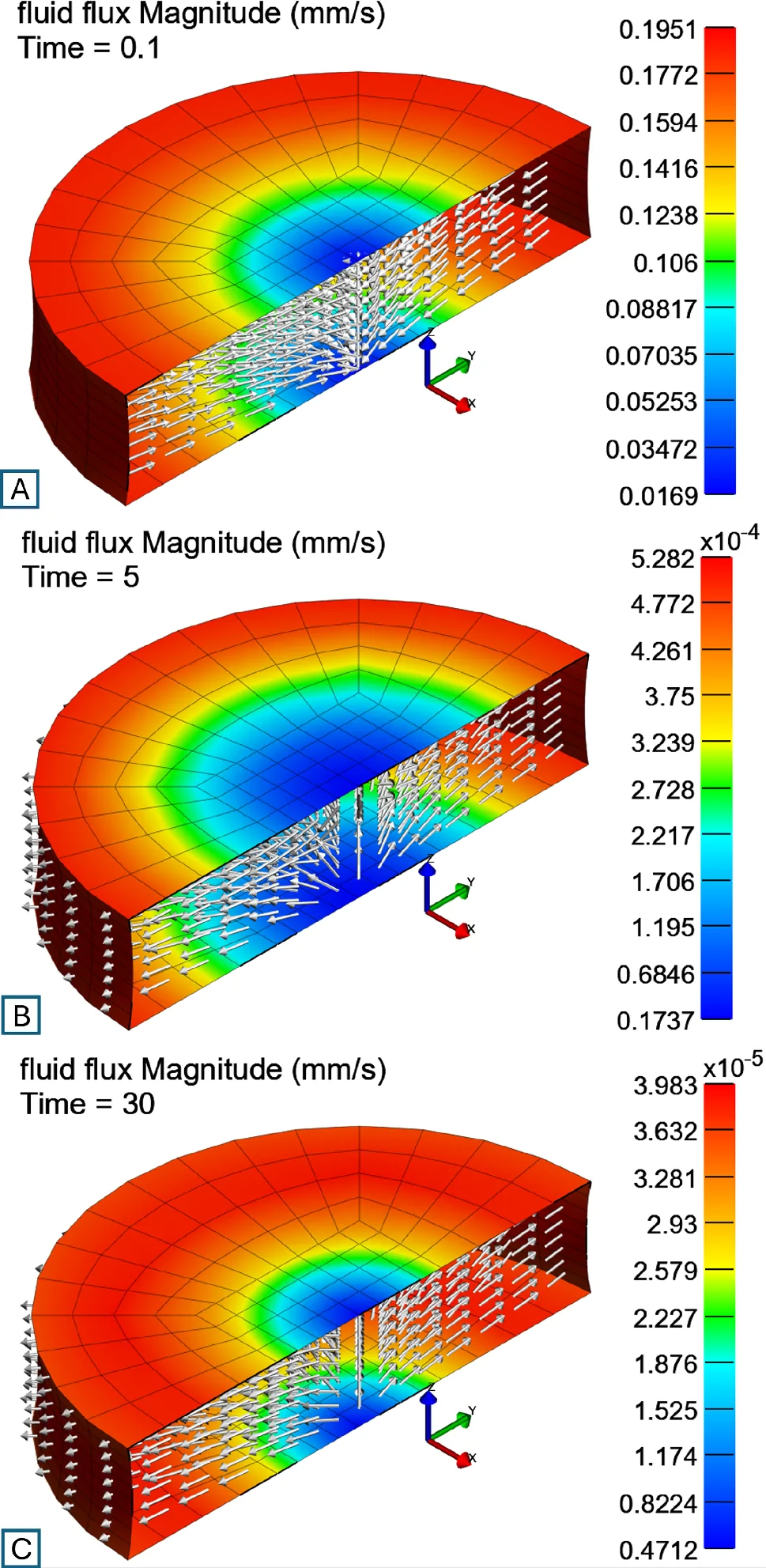
Spatio-temporal evolution of fluid flux across the specimen cross-section. The contour plots illustrate the fluid velocity magnitude (mm/s), while white arrows represent the flux vectors. **A** At t=0.1 s, inward vectors show the initial suction effect (swelling) driven by the rapid radial expansion. **B,C** At t=5 s and t=30 s, the reversal of vector direction shows the outward fluid exudation (relaxation phase), characterized by a decay in flux magnitude from $10^{-4}$ to $10^{-5}$ mm/s. This transition highlights the role of fluid redistribution in the overall stress-relaxation process

To isolate the fluid’s contribution to the transient response before the onset of microstructural damage, the spatial and temporal evolution of interstitial fluid dynamics was analyzed during the initial relaxation step, focusing on the small-strain regime despite the large deformations (>200%) reached experimentally (Fig. 8). The primary variable examined is the fluid flux magnitude, which represents the volumetric flow rate of the interstitial fluid per unit area of the porous medium (mm/s). In the biphasic framework, this variable quantifies the rate at which the fluid is redistributed or exuded due to the pressure gradients generated by the radial stretching of the solid matrix.

The simulation reveals a dynamic inversion of the fluid flux direction within the 30-second interval. At the onset of loading (t=0.1 s, Fig. 8A), the flux vectors are directed inward toward the specimen core. This phenomenon indicates an instantaneous expansion (swelling) of the porous matrix, where the rapid increase in pore volume generates a localized suction that draws fluid from the boundaries to reach the initial peak stress. As the relaxation phase progresses (t=5 s, Fig. 8B), when the reaction force has already decayed by approximately 70%, the flow direction reverses. The vectors point outward, indicating that the interstitial fluid is being exuded from the core toward the lateral boundaries. Although the flux magnitude drops significantly (order of $10^{-4}$ mm/s), this outward redistribution is essential for the dissipation of pore pressure gradients. At the end of the step (t=30 s, Fig. 8C), the fluid continues to exit the specimen with even lower velocities (order of $10^{-5}$ mm/s) distributed uniformly across the geometry. This temporal shift from inward suction to outward exudation confirms that the initial transient response is governed by the complex interplay between the volumetric expansion of the matrix and the subsequent poroelastic equilibration.

This high level of correlation, combined with the consistent fluid-dynamic behavior, confirms that the model implementation within FEBio accurately solves the coupled poromechanical equations. By demonstrating that the numerical solution is faithful to the analytical constitutive expression, this validation provides a verified and reliable foundation for future complex structural simulations involving heterogeneous geometries or non-uniform loading conditions.

## Discussion

### Radial tensile testing as a relevant mechanical proxy for aortic dissection

The present results confirm that radial tensile testing provides a mechanically relevant framework for investigating damage mechanisms associated with aortic dissection. While traditional uniaxial tests in the axial or circumferential directions probe the high-stiffness collagen networks (often yielding stresses in the MPa range), radial loading specifically targets the interlamellar cohesion of the medial layer (Ferrara et al. 2016). The findings of remarkably low stiffness (order of kPa) and the progressive loss of integrity at relatively low force levels explain the low resistance to dissection propagation observed clinically (Sommer et al. 2010). By isolating the out-of-plane mechanical properties, this methodology complements classical tensile tests and provides the necessary data to understand why the aorta, despite its strength in-plane, remains highly vulnerable to delamination once an initial intimal tear occurs (Sherifova and Holzapfel 2019).

### Role of fluid–solid interactions under radial loading

A significant insight from our results is that the observed stress relaxation cannot be explained by solid-phase viscoelasticity alone. The biphasic formulation proved essential to capture the initial transient response and the subsequent relaxation kinetics (Simon 1992). This is consistent with the foundational theories of hydrated biological tissues, where interstitial fluid pressurization functions as a primary load-support mechanism (Shim et al. 2021). As shown by the numerical analysis in Fig. 8, the poromechanical state of the tissue undergoes a dynamic evolution during the first 30 s of relaxation. These results demonstrate that stress relaxation is a multi-physics process where the biphasic coupling dictates the initial transient (Bukac et al. 2015). At the onset of loading (t=0.1 s), the rapid interlamellar expansion generates a negative pressure gradient, triggering an instantaneous inward flux (suction) that contributes to the peak engineering stress. This phenomenon is exacerbated by the hierarchical organization of the lamellar units, which act as semi-permeable barriers to rapid fluid flow, effectively trapping the fluid within the interlamellar spaces (Denny et al. 2012). As relaxation progresses (t=5 s), a direction inversion occurs: the fluid begins to exit the boundaries (exudation), with flux magnitudes decaying from $10^{-1}$ to $10^{-4}$ mm/s. This transition marks the dissipation of the pore pressure, changing the response from poro-viscoelastic coupling to the relaxation of the purely intrinsic solid matrix (Ateshian 2009). By t=30 s, the residual exudation ($10^{-5}$ mm/s) confirms poromechanical equilibration. This suggests that under rapid loading conditions, where fluid redistribution cannot occur instantaneously, the trapped interstitial fluid generates high localized pressures that force the lamellae apart, accelerating the microstructural damage leading to aortic delamination.

### Progressive damage as a key mechanism preceding dissection

The identification of the damage variable *D* demonstrates that microstructural degradation in the aorta is a gradual, history-dependent process rather than a sudden rupture (Volokh 2022). The observed plateauing of stress at high radial stretch represents the onset of micro-tears and the loss of interlamellar cross-links (Samila and Carter 1981). The microstructural evidence from two-photon microscopy supports this: the extreme reorientation of fibers toward $95^\circ$ implies a severe reorganization that likely precedes visible mechanical failure. It is important to emphasize that damage is not synonymous with rupture; rather, damage constitutes the accumulation of microstructural defects that weakens the tissue, creating a predisposed plane for the infiltration of blood and the subsequent formation of a false lumen (Rolf-Pissarczyk et al. 2021; Holzapfel and Ogden 2025). Such microstructural precursors are consistent with the progressive failure of elastic lamellae observed in longitudinal studies of medial degeneration (Witzenburg et al. 2017). To validate the physical consistency of the identified material parameters, the radial failure metrics were correlated with the classic direct tension and peeling data on aortic media reported by Sommer et al. (2008). The ultimate radial stress peak captured by the constitutive framework is in close agreement with their reported direct radial tensile strength of 140±15.9 kPa. Furthermore, the continuous log-normal formulation successfully captures the progressive transition between the elastic state and the micro-defect accumulation state, mirroring the experimental S1-S2 phase transition documented during out-of-plane loading. Finally, the localized damage accumulation $D_\text {max}\approx 0.60$ provides a representative description of the degraded material state within the 6–7 elastic lamellae thickness that forms the cohesive zone at the propagation front during steady-state tissue peeling. This close multi-scale correlation confirms that the proposed constitutive parameters possess a clear, verifiable physical meaning tied to the macroscopic mechanics of delamination.

### Novelty and positioning relative to existing models

The proposed framework fills a critical gap in the current biomechanical literature. Most existing constitutive models (e.g., Holzapfel et al. (2000); Gasser et al. (2006)) are specifically designed for axial and circumferential data and frequently neglect biphasic effects or assume perfect interlamellar bonding (Ferrara et al. 2016; Balzani et al. 2012). While those models are excellent for predicting burst pressure, they lack validation and sensitivity under radial tension. This work is specifically designed for radial tensile tests and successfully integrates biphasic, viscoelastic, and damage effects into a single, FEBio-validated implementation. The choice of a reactive framework is particularly suited for biological tissues where continuous remodeling of internal bonds occurs under load. By utilizing imaging-based priors for fiber architecture, we ensure that the model parameters represent the actual physical state of the tissue, consistent with structural modeling approaches that emphasize the role of discrete fiber orientations (Balzani et al. 2012). This reduces the mathematical ambiguity of the fit, ensuring that the results are not merely numerical artifacts. Furthermore, while the current work adopts a structural description rooted in the classical Holzapfel-Gasser-Ogden (HGO) framework, alternative advanced structural paradigms have recently emerged to overcome the limitations of partitioning the extracellular matrix into discrete fiber families. Specifically, the newly developed Unified-Fiber-Distribution (UFD) model treats the structural network as a continuous, unified planar distribution (Dong and Sun 2021). This approach offers superior physical consistency with real tissue morphology and naturally captures complex multi-axial phenomena, such as the second kind of Poisson effect (Dong et al. 2023). Integrating the UFD framework within the proposed reactive viscoelastic and biphasic description represents a promising avenue for future developments. In such an implementation, the continuous angular fiber density function would be embedded directly within the hereditary time-integrals, allowing the model to track the simultaneous viscoelastic relaxation and progressive damage of the continuous network orientations. Mechanistically, the UFD-coupled kinematics would link lateral matrix contractions directly to the pore-fluid exudation rate and localized shear states, potentially providing an even more refined prediction of the multi-physical precursors that govern interlamellar delamination.

### Limitations and perspectives

Despite the robustness of the model, some limitations must be acknowledged. The experimental setup utilizes a uniaxial radial idealization, whereas the in-vivo stress state is multiaxial. Specifically, another idealization involves adopting a localized uniaxial strain condition, assuming zero lateral deformation despite the free boundaries and high cumulative stretches of the specimens. Nonetheless, this kinematic simplification is regularized by the tissue’s multi-physics nature and the constitutive framework. Instantaneous poromechanical confinement of the incompressible fluid and the high in-plane stiffness of the collagen network physically resist short-term lateral contraction. Furthermore, because the reactive formulation operates on the relative deformation gradient within small incremental steps (∼4% strain), the kinematics within each relaxation window remain infinitesimal, minimizing global geometric errors. Future multi-axial models will relax this assumption to capture non-uniform stress states across complex vascular geometries.

Additionally, the scalar damage law assumes a homogenized degradation within the sample, whereas aortic dissection is often characterized by localized failure between specific lamellar units.

Similarly, the fiber orientation was embedded using a standard affine framework based on a constant reference angle γ. While this approach successfully captures the deformation-induced reorientation, the calibration is tailored to out-of-plane radial loading. This limits the direct transferability of the identified material parameters to other loading histories, such as in-plane biaxial testing or inflation configurations, where direct in-plane fiber recruitment dominates the macroscopic mechanical response.

Looking forward, integrating this radial framework into large-scale, patient-specific finite element simulations represents a natural extension of this work. Within an orthotropic formulation, the reactive viscoelastic-damage model can be combined with standard circumferential and axial HGO fiber descriptions. While the current damage law is driven by the total strain energy density, large-scale multi-axial simulations will leverage an energy-splitting scheme. By selectively coupling the damage variable *D* to the volumetric and matrix-shear energy potentials, degradation will be limited to the interlamellar space. This prevents non-physical cross-degradation of the in-plane structural properties during multi-axial inflation, ensuring accurate predictions of radial delamination and false lumen propagation without compromising global vessel stability.

The transition from ex-vivo porcine data to in-vivo human pathology will require correlating these mechanical parameters with histological markers of aging and disease, as suggested by microstructurally motivated models of arterial remodeling (Bellini et al. 2014). Nonetheless, the current model provides a verified tool for studying the interplay between fluid flow and tissue damage, offering new insights into the mechanical precursors of aortic delamination.

## Data Availability

No datasets were generated or analysed during the current study.

## Appendix A Individual specimen calibrations

To ensure full transparency and complement the averaged constitutive properties presented in Sect. 3.3, the individual material parameters and corresponding goodness-of-fit metrics ($R^2$ and *RMSE*) identified for each independent porcine specimen (n=5) are detailed in Table 3.

**Table 3:** Individual specimen parameter sets, fixed microstructural priors and constitutive fit quality metrics

Specimen	*E* (kPa)	ν	*c* (kPa)	$k_1$ (kPa)	$k_2$	τ (s)	μ (kPa)	σ	$D_{\max }$	$R^2$	RMSE (kPa)
Specimen 1	10.526	0.490	2.53	7.60	1.810	10.83	3.50	0.60	0.56	0.93	3.01
Specimen 2	10.514	0.330	2.40	8.57	1.809	1.86	3.50	0.60	0.64	0.96	1.39
Specimen 3	10.534	0.496	2.62	6.89	1.811	13.20	3.50	0.60	0.50	0.87	3.44
Specimen 4	10.521	0.480	2.47	9.01	1.809	5.64	3.50	0.60	0.69	0.98	2.07
Specimen 5	10.530	0.490	2.58	8.13	1.811	8.47	3.50	0.60	0.61	0.91	2.59
Mean	10.525	0.490	2.52	8.04	1.810	8.00	(Fixed)	(Fixed)	0.60	0.93	2.50
Std. Dev	0.008	0.150	0.09	0.83	0.001	4.43	–	–	0.07	0.04	0.80

## Appendix B Numerical analysis–constitutive relation

Using Wolfram Mathematica software, the following constitutive relation of the reactive viscoelastic material has been identified. Two different constitutive models were chosen for the strong and weak bonds: Neo-Hookean Material for the strong bonds and HGO Unconstrained Material for the weak bonds.

### B.1 Kinematics: uniaxial strain condition

Based on the boundary conditions of the radial tensile test - where lateral expansions in the *x* and *y* directions are constrained - the deformation gradient $\textbf{F}(t)$ simplifies to a uniaxial strain state:

B1 $$\begin{aligned} \textbf{F}(t) = \begin{pmatrix} 1 & 0 & 0 \\ 0 & 1 & 0 \\ 0 & 0 & \lambda _z(t) \end{pmatrix} \end{aligned}$$

Consequently, the left Cauchy-Green tensor $\textbf{B} = \textbf{F}\textbf{F}^T$ and the kinematic invariants are derived as:

B2 $$\begin{aligned} J&= \det (\textbf{F}) = \lambda _z \end{aligned}$$

B3 $$\begin{aligned} \textbf{B}&= \text {diag}(1, 1, \lambda _z^2)\end{aligned}$$

B4 $$\begin{aligned} I_1&= \text {tr}(\textbf{B}) = 2 + \lambda _z^2 \end{aligned}$$

### B.2 Strong bonds–Neo-Hookean

The Cauchy stress tensor $\boldsymbol{\sigma }^e$ for an isotropic hyperelastic material is obtained from the strain energy density through the following relationship:

B5 $$\begin{aligned} \boldsymbol{\sigma }^e = \frac{1}{J} \left[ 2 \frac{\partial \Psi ^e}{\partial I_1} \textbf{B} + J \frac{\partial \Psi ^e}{\partial J} \textbf{I} \right] \end{aligned}$$

Differentiating Eq. 4 with respect to the invariants yields:

B6 $$\begin{aligned} \frac{\partial \Psi ^e}{\partial I_1}&= \frac{\mu _e}{2} \end{aligned}$$

B7 $$\begin{aligned} \frac{\partial \Psi ^e}{\partial J}&= -\frac{\mu _e}{J} + \frac{\lambda _{e}}{J} \ln J \end{aligned}$$

Substituting these derivatives into Eq. B5, the explicit form of the stress tensor is obtained:

B8 $$\begin{aligned} \boldsymbol{\sigma }^e = \frac{\mu _e}{J} (\textbf{B} - \textbf{I}) + \frac{\lambda _{e}}{J} (\ln J) \textbf{I} \end{aligned}$$

By extracting the radial component $\sigma _{zz}^e$ and substituting the kinematic relations $J = \lambda _z$ and $B_{zz} = \lambda _z^2$, the final expression for the radial stress is:

B9 $$\begin{aligned} \sigma _{zz}^e (\lambda _z) = \underbrace{\frac{\mu _e}{\lambda _z} (\lambda _z^2 - 1)}_{\text {Distortional response}} + \underbrace{\frac{\lambda _{e}}{\lambda _z} \ln (\lambda _z)}_{\text {Volumetric response}} \end{aligned}$$

The relationship between the material parameters, E, and ν, and the parameters used in the strain-energy function, is as follows:

B10 $$\begin{aligned} \mu _e = \frac{E}{2(1 + \nu )} \quad ; \quad \lambda _e = \frac{\nu E}{(1 + \nu )(1 - 2\nu )} \end{aligned}$$

### B.3 Weak bonds—Holzapfel Gasser Ogden model

The transient weak bonds are described by the Holzapfel-Gasser-Ogden (HGO) unconstrained formulation. Unlike the perfectly incompressible case, this model accounts for the volumetric changes associated with pore dilation in the biphasic framework. The strain energy density $\Psi _r^b$ for a bond generation formed at time *u* is given by:

B11 $$\begin{aligned} \Psi _r^b = \Psi _{matrix}^b + \Psi _{fibers}^b + \Psi _{vol}^b \end{aligned}$$

#### B.3.1 Kinematics and invariants

Under the prescribed uniaxial strain condition ($\lambda _x = \lambda _y = 1$), the relative deformation gradient for a generation born at time *u* is $\textbf{F}^u ={diag} (1, 1, \lambda _{rel})$, where $\lambda _{rel} = \lambda _t / \lambda _u$. The Jacobian and the relevant invariants of the relative right Cauchy-Green tensor $\textbf{C}_u$ are derived as:

B12 $$\begin{aligned} J_u&= \det (\textbf{F}^u) = \lambda _{rel}\end{aligned}$$

B13 $$\begin{aligned} I_1&= {tr}(\textbf{C}_u) = 2 + \lambda _{rel}^2\end{aligned}$$

B14 $$\begin{aligned} I_{4\alpha }&= \textbf{a}_\alpha \cdot \textbf{C}_u \cdot \textbf{a}_\alpha \end{aligned}$$

The two symmetric families of collagen fibers lie entirely within the tangential plane of the aortic media (the local ${e_1, e_2}$ plane), which is perpendicular to the radial loading axis *z* (${e_3}$). Their direction vectors in the reference configuration are defined as $\textbf{a}_{1,2} = [\cos \gamma , \pm \sin \gamma , 0]^T$, where $\gamma = 60^\circ$ is the fixed in-plane mean orientation angle relative to the circumferential axis. The structural invariants $I_{4\alpha }$ are explicitly resolved via tensor projection:

B15 $$\begin{aligned} & I_{4\alpha } = \textbf{a}_{\alpha } \cdot \textbf{C}_u \cdot \textbf{a}_{\alpha } = \begin{pmatrix} \cos \gamma&\pm \sin \gamma&0 \end{pmatrix}\nonumber \\ & \begin{pmatrix} 1 & 0 & 0 \\ 0 & 1 & 0 \\ 0 & 0 & \lambda _{\text {rel}}^2 \end{pmatrix} \begin{pmatrix} \cos \gamma \\ \pm \sin \gamma \\ 0 \end{pmatrix} = \cos ^2\gamma + \sin ^2\gamma = 1 \end{aligned}$$

#### B.3.2 Constitutive equations

Consistent with the FEBio implementation for HGO unconstrained materials, the individual energy terms are defined as:

B16 $$\begin{aligned} \Psi _\text {matrix}^b&= \frac{c}{2}(I_1 - 3 - 2 \ln J_u) \end{aligned}$$

B17 $$\begin{aligned} \Psi _\text {fibers}^b&= \frac{k_1}{2k_2} \sum _{\alpha =1}^2 \left( \exp \left( k_2 \langle E_\alpha \rangle ^2 \right) - 1 \right) \end{aligned}$$

B18 $$\begin{aligned} \Psi _\text {vol}^b&= \frac{K}{2} \left( \frac{J_u^2 - 1}{2} - \ln J_u \right) \end{aligned}$$

where *c* is the matrix shear modulus, $k_1, k_2$ are fiber stiffness parameters, and *K* is the bulk modulus. The fiber strain $E_\alpha$ accounts for the structural dispersion κ:

B19 $$\begin{aligned} E_\alpha = \kappa (I_1 - 3) + (1 - 3\kappa )(I_{4\alpha } - 1) \end{aligned}$$

Substituting $I_{4\alpha } = 1$ into the HGO fiber strain formulation, the generalized fiber strain simplifies to a form exclusively driven by the structural dispersion κ:

B20 $$\begin{aligned} E_{\alpha } = \kappa (I_1 - 3) = \kappa (\lambda _{{rel}}^2 - 1) \end{aligned}$$

#### B.3.3 Derivation of the radial Cauchy stress

The radial Cauchy stress $\sigma _{zz}^{b}$ is obtained by differentiating the energy potential with respect to the relative stretch. Given the uniaxial kinematics, the stress simplifies to $\sigma _{zz}^b = \partial \Psi _r^b / \partial \lambda _{rel}$. Differentiating each contribution yields:

- **Matrix stress**
  B21 $$\begin{aligned} \frac{\partial \Psi _\text {matrix}^b}{\partial \lambda _\text {rel}} = \frac{c}{\lambda _\text {rel}}(\lambda _{rel}^2 - 1) \end{aligned}$$
- *Fiber stress* By defining the auxiliary function $S_\text {fib} = k_1 \langle E_\text {fib} \rangle \exp (k_2 \langle E_\text {fib} \rangle ^2)$ and noting the symmetry of the fiber families ($E_1 = E_2 = E_\text {fib}$), the contribution is:
  B22 $$\begin{aligned} \sigma _\text {fiber} = 4 S_\text {fib}(\lambda _\text {rel}) \left[ \kappa \lambda _\text {rel} + (1-3\kappa )\lambda _\text {rel} \sin ^2\theta \right] \end{aligned}$$
- **Volumetric stress**
  B23 $$\begin{aligned} \frac{\partial \Psi _\text {vol}^b}{\partial \lambda _\text {rel}} = \frac{K}{2\lambda _\text {rel}}(\lambda _\text {rel}^2 - 1) \end{aligned}$$

Combining these terms, the final expression for the radial stress component of the HGO weak bonds is:

B24 $$\begin{aligned} & \sigma _{zz}^{b}(\lambda _\text {rel}) = \frac{c + \frac{K}{2}}{\lambda _\text {rel}}(\lambda _\text {rel}^2 - 1) + 4 S_\text {fib}(\lambda _{rel})\nonumber \\ & \left[ \kappa \lambda _\text {rel} + (1-3\kappa )\lambda _\text {rel} \sin ^2\theta \right] \end{aligned}$$

This formulation allows the model to capture the non-linear recruitment of collagen fibers during radial expansion. Within the biphasic framework, the $J_u$-dependent terms in Eq. B24 represent the mechanical resistance of the transient solid phase to pore volume changes, directly influencing the interstitial fluid pressure and the overall relaxation behavior of the aortic tissue.

## Appendix C Mathematical specification of the reactive damage framework

To ensure full reproducibility and complement the constitutive equations described in Sect. 2.6.2, this section details the continuum reactive damage framework implemented within the FEBio environment to model out-of-plane tissue softening and interlamellar failure. The microstructural damage process is assumed to be driven by the deformation of the intact solid matrix. The scalar damage criterion measure, Ξ, is defined directly as the strain energy density function of the undamaged, intact material configuration ($\Psi _0$):

C25 $$\begin{aligned} \Xi (\textbf{C}_u) = \Psi _0(\textbf{C}_u) = \Psi _{\text {matrix}}^0(\textbf{C}_u) + \Psi _{\text {fibers}}^0(\textbf{C}_u) \end{aligned}$$

where $\textbf{C}_u = \textbf{F}_u^T \textbf{F}_u$ represents the relative right Cauchy-Green deformation tensor, which measures the strain sustained by the specific generation of structural bonds relative to their reference configuration at the time of birth *u*. Concurrently, $\Psi _{\text {matrix}}^0$ and $\Psi _{\text {fibers}}^0$ represent the strain energy contributions of the intact isotropic ground substance and the anisotropic fiber families, respectively.

To satisfy thermodynamic consistency, guarantee path-dependency, and enforce the physical irreversibility of tissue degradation during arbitrary loading histories, the active driving quantity $\Xi _m(t)$ is defined as the historical maximum of the strain energy density achieved from the initial undamaged state up to the current simulation time *t*:

C26 $$\begin{aligned} \Xi _m(t) = \max _{-\infty < s \le t} \Psi _0\big (\textbf{C}_u(s)\big ) \end{aligned}$$

This condition strictly ensures that further microstructural damage accumulates ($\dot{D}> 0$) if and only if the current energy state exceeds the previous historical threshold ($\Xi = \Xi _m$ and $\dot{\Xi }> 0$); otherwise, the damage parameter remains stationary ($\dot{D} = 0$).

### Log-normal damage evolution profile

The progressive mass fraction of ruptured interlamellar connections and structural bonds (*D*) is governed by a three-parameter log-normal cumulative distribution function (CDF), regularized and scaled by the ultimate maximum damage limit ($D_{\max }$):

C27 $$\begin{aligned} D(\Xi _m) = D_{\max } \cdot \mathcal {F}(\Xi _m; \mu , \sigma ) \end{aligned}$$

Expressing the log-normal cumulative distribution analytically via the complementary error function ($\text {erfc}$) yields the explicit computational form implemented in the FEBio solver:

C28 $$\begin{aligned} D(\Xi _m) = D_{\max } \cdot \frac{1}{2} \text {erfc}\left( - \frac{\ln \left( \frac{\Xi _m}{\mu }\right) }{\sigma \sqrt{2}} \right) \end{aligned}$$

where the constitutive parameters possess distinct physical interpretations:

- μ [$\text {kPa}$] is the median location parameter, which represents the critical strain energy density threshold at which exactly half of the available structural interlamellar links undergo failure ($D = 0.5 \cdot D_{\max }$).
- σ [dimensionless] is the continuous spread parameter, regulating the standard deviation and the slope of the softening phase, thus controlling how rapidly damage propagates as the strain energy increases.
- $D_{\max }$ [dimensionless, $0 \le D_{\max } \le 1$] represents the specimen-specific asymptotic saturation ceiling of damage, quantifying the ultimate mass fraction of permanent structural cohesion lost by the tissue volume prior to elements elimination.

### Microstructural priors and parameter identifiability

Simultaneously treating $D_{\max }$, μ, and σ as free parameters during inverse numerical optimization yields severe parameter cross-correlations and optimization non-uniqueness (over-parameterization), where the numerical solver can trade off a horizontal shift in μ for a change in the slope σ. To address this mathematical identifiability challenge, a two-step calibration strategy was adopted. Preliminary baseline screenings on a subset of the experimental curves established that damage initiation for the porcine aortic media consistently centers around an energy density of $\mu = 3.50\text { kPa}$, with a smooth, continuous transition envelope described by σ=0.60. Locking these values as fixed microstructural priors for the entire tissue cohort stabilizes the inverse problem, avoiding overfitting while isolating $D_{\max }$ as the sole active free parameter capturing inter-specimen structural variability.
